# Therapeutic remodeling of the tuberculosis granuloma with 1-methyl-D-tryptophan enhances CD8^+^ T cell–macrophage interactions

**DOI:** 10.1073/pnas.2528104123

**Published:** 2026-08-11

**Authors:** Erin F. McCaffrey, Alea C. Delmastro, Bindu Singh, Annu Devi, Caden W. Munson, Priyanka, Nadia A. Golden, Shabaana A. Khader, Michael Angelo, Deepak Kaushal, Smriti Mehra

**Affiliations:** ^a^ Department of Pathology, Stanford University School of Medicine, Stanford, CA 94305; ^b^ Spatial Immunology Unit, Laboratory of Parasitic Diseases, National Institute of Allergy and Infectious Diseases, National Institutes of Health, Bethesda, MD 20892; ^c^ Texas Biomedical Research Institute, San Antonio, TX 78245; ^d^Tulane National Primate Research Center, Division of Bacteriology and Parasitology, Covington LA 70433; ^e^ Department of Microbiology and Immunology, University of Chicago School of Medicine, Chicago, IL 60637

**Keywords:** multiplexed imaging, *Mycobacterium tuberculosis*, granuloma, macaque

## Abstract

Granulomas, the hallmark of tuberculosis (TB) disease, can both restrict *Mycobacterium tuberculosis* (*Mtb*) dissemination and impede its clearance. Recent studies indicate that indoleamine 2,3-dioxygenase (IDO1), an immunosuppressive metabolic enzyme, limits infiltration of activated T cells and can contribute to TB disease progression. Treatment with 1-methyl-D-tryptophan (D-1MT), a small molecule inhibitor that restores mTOR signaling, has been shown to reduce IDO1 activity and improve immune responses in *Mtb*-infected rhesus macaques. Here, we investigated the impact of D-1MT treatment on TB granuloma architecture using 30-plex high-dimensional issue imaging in rhesus macaques. By spatially mapping 13 distinct cell populations, we found D-1MT treatment corresponded with significantly increased infiltration CD8^+^ T cells into granulomas compared to untreated controls. Notably, these CD8^+^ T cells expressed markers of cell proliferation and cytotoxicity. D-1MT enhanced CD8^+^ T cell infiltration throughout the granuloma, with particularly pronounced effects in the myeloid core, where we observed significantly enhanced spatial interactions between macrophages and CD8^+^ T cells, but not CD4^+^ T cells. Our results demonstrate that: i) effective intragranulomatous *Mtb* control is associated with the close spatial proximity between CD8^+^ T cells and macrophages, a feature less abundant in uncontrolled pulmonary TB; ii) IDO1 induction blocks CD8^+^ T cell infiltration and reduces T cell activation and proliferation; and iii) therapeutic strategies, including D-1MT, that improve intragranulomatous killing hold strong translational potential.

## Significance

Our understanding of immune mechanisms within the tuberculosis (TB) granuloma has advanced greatly with the advent of high-resolution single-cell multiplexed imaging. Using such imaging, we show that TB granulomas in rhesus macaques, a highly translational model of human TB pathology, are characterized by indoleamine 2,3-dioxygenase 1 (IDO1)-mediated immunoregulation. Early pharmacologic restoration of mTOR signaling via 1-methyl-D-tryptophan (D-1MT) treatment can reduce IDO1 enzymatic activity and facilitate the recruitment and function of CD8^+^ T cells within the granulomas. These findings reveal specific mechanisms exploited by *Mycobacterium tuberculosis* (*Mtb*) to maintain intragranuloma persistence and underscore immune responses. Future vaccine and therapeutic design should consider these immunoregulatory features to achieve better control of *Mtb* infection.

*Mycobacterium tuberculosis* (*Mtb*) is the leading cause of mortality from infectious disease worldwide, accounting for nearly 1.5 million deaths each year. Relative to other infectious diseases, the reduction in the incidence of tuberculosis (TB) disease over the last 20 y has been unimpressive (1). This is largely due to the continued lack of a highly efficacious vaccine, lengthy and toxic antimicrobial regimens, and emergence of multidrug resistance. Along these lines, efforts to develop new host-directed therapies for TB will require a deeper understanding of interactions between *Mtb* and the human immune system (2).

The formation of granulomas is a hallmark of *Mtb* infection. A prototypical granuloma consists of a myeloid-predominant central core region that is highly enriched in infected macrophages and neutrophils and encircled by lymphocytes (3). These lesions are also characterized by the presence of variable levels of viable *Mtb*, necrosis, or cell death, fibrosis, and immune activation (4). From the perspective of facilitating an effective host response, granulomas play central and seemingly contradictory roles. On one hand, granulomas can sequester *Mtb* and limit dissemination to uninfected tissue sites. On the other hand, upregulated tolerogenic pathways within the myeloid core may limit bacterial clearance, and granuloma-associated inflammation can cause damaging host pathology (5).

The nonhuman primate (NHP) model of TB—using experimentally infected rhesus (RM) or cynomolgus (CM) macaques—has been shown to recapitulate the functional diversity of the human TB granuloma (6). Work in these model systems has demonstrated that granulomas within a single *Mtb-*infected individual can take on a spectrum of fates from complete bacterial clearance to uncontrolled dissemination and inflammation (7–9). This strongly suggests that local host-bacterial dynamics within an individual granuloma’s microenvironment govern granuloma function and that granuloma structure and immune cell function are interconnected. These data also suggest that, while some granulomas pose a barrier to controlling *Mtb* infection, others do in fact have the cellular and organizational requirements for bacterial control.

We have previously demonstrated that one of the most highly abundant proteins in both human and NHP TB granulomas is indoleamine 2,3-dioxygenase 1 (IDO1) (5, 10). IDO1 is a metabolic enzyme that catalyzes the conversion of tryptophan to kynurenine (11). Depletion of tryptophan by IDO1 has been shown to be protective against pathogens, but it also has highly immunosuppressive effects on macrophages and T cells (12, 13). In human TB, IDO1 is expressed by granuloma macrophages alongside other suppressive attributes, such as programmed death ligand 1 (PD-L1), TGFβ, and regulatory T cells (Tregs) (5). Because we hypothesize IDO1 is suppressing anti-*Mtb* immunity in the granuloma, it is a potential target for host-directed immunotherapy.

One candidate approach for targeting IDO1’s activity is the small molecule 1-methyl-D-tryptophan (D-1MT). D-1MT acts as a mimetic of the amino acid tryptophan, thus reversing the inhibitory impact of tryptophan depletion (14). Recent work suggests the mechanism of D-1MT is primarily through restoration of mTOR signaling, which is critical for T cell effector functions (14, 15). We have demonstrated that use of this small molecule in vivo can reduce IDO1 enzymatic activity in RMs, enhance innate and adaptive immune responses, and reorganize the granuloma to provide T cells greater access to the granuloma core in RM lungs (10, 16). Given its potential as a host-directed therapy for TB, we sought to more deeply characterize the impact of D-1MT treatment on TB granuloma composition and structure. Here, we used Multiplexed Ion Beam Imaging by Time-of-Flight (MIBI-TOF) to image 30 proteins at subcellular resolution and spatially map 13 cell subsets in TB granulomas from RMs treated with D-1MT or those left untreated. Our results first confirm that TB granulomas in both RMs and humans share an immunoregulatory myeloid core characterized by abundant IDO1. Furthermore, we find that D-1MT treatment is associated with a substantial increase specifically in CD8^+^ (but not CD4^+^) T cell infiltration and proliferation in the myeloid core. This suggests that inhibition of IDO1 preferentially aids in CD8^+^ T cell immunity in the TB granuloma. Ultimately, this study explores the role of D-1MT as an immunotherapeutic strategy to enhance cellular immunity in granulomas during *Mtb* infection.

## Results

### Application of MIBI-TOF Platform to RM Tissues and Benchmarking with Human TB Granulomas.

**Fig. 1.**
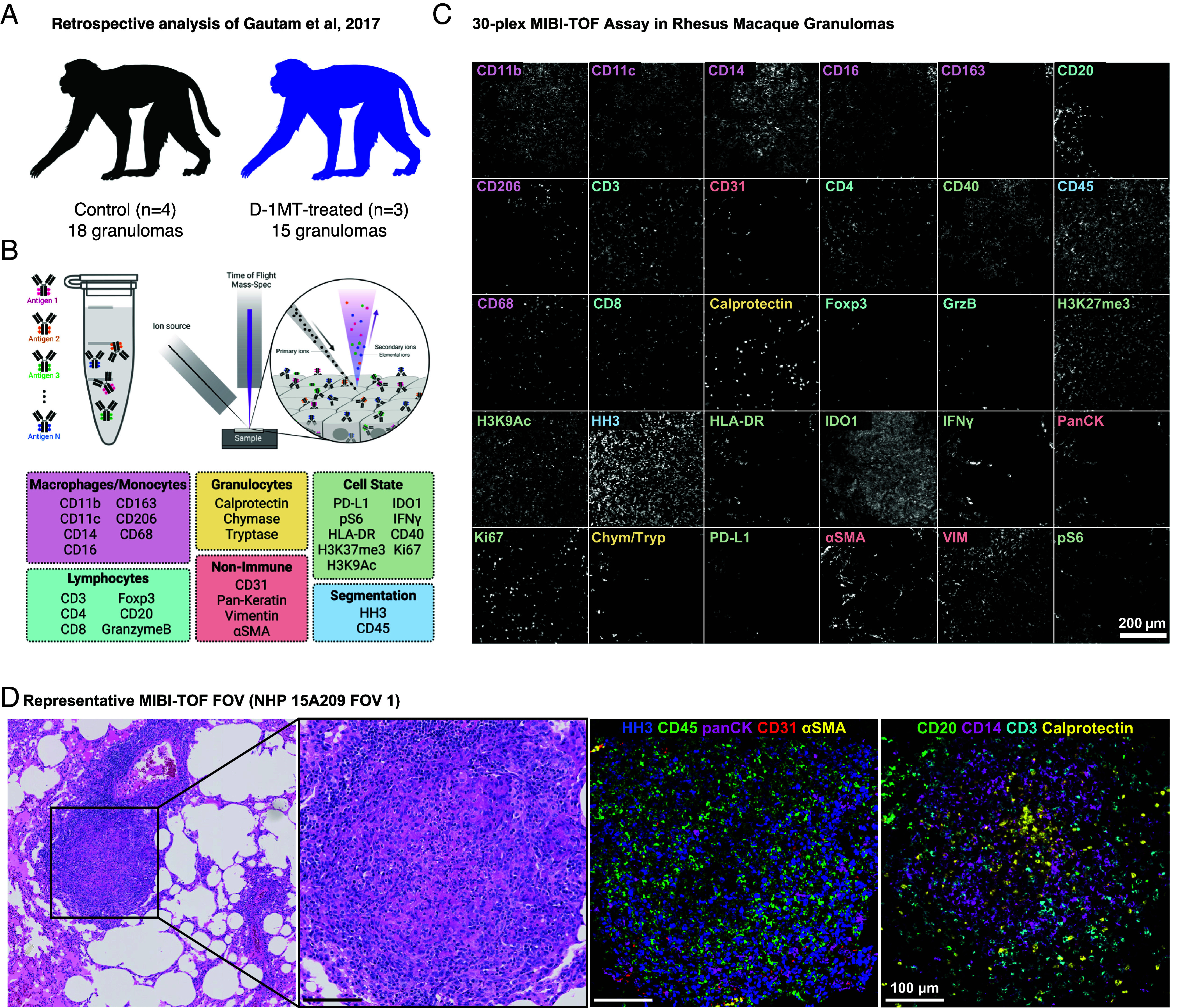
Study design. (*A*) Cohort characteristics, including the number of RMs and the total number of granulomas analyzed by the treatment group. Biopsy specimens were obtained from Gautam et al. (10). (*B*) Graphical illustration of MIBI-TOF methodology (*Top*) and the list of 31 markers included in the imaging panel (*Bottom*). (*C*) Expression patterns for the 30 marker channels acquired for one representative granulomatous FOV. (*D*) One representative hematoxylin & eosin–stained specimen with *Inset* demonstrating FOV acquired on MIBI-TOF. On the right, two MIBI-TOF overlays, demonstrating major lineage markers: (Third from left) αSMA (yellow), panCK (magenta), CD45 (green), HH3 (blue), and CD31 (red); (Fourth from left) Calprotectin (yellow), CD14 (magenta), CD20 (green), and CD3 (cyan). (Scale bar: 100 μm unless otherwise indicated.)

We performed an in-depth investigation into the spatial biology of pulmonary TB granulomas in RMs with or without concurrent D-1MT treatment. For these analyses, we curated a cohort of archival formalin-fixed paraffin-embedded (FFPE) lung sections from seven RMs infected with a high dose (~100 to 200 CFU) of *Mtb* CDC1551 via the aerosol route that resulted in acute pulmonary TB (10). Of the seven *Mtb*-infected RMs included in this study, four RMs were untreated (henceforth referred to as controls), and three RMs were treated with D-1MT (45 mg/kg body weight) orally daily, starting 1 wk after *Mtb* infection, as previously reported (10). All animals were necropsied at weeks 5 to 8 postinfection, which allows us to characterize early TB granuloma composition and phenotype. In total, our imaging cohort comprised 500 × 500 μm fields-of-view (FOVs), capturing 18 pulmonary granulomas from four control RMs and 15 pulmonary granulomas from three D-1MT-treated RMs (Fig. 1*A* and *SI Appendix*, Fig. S1).

All tissues were analyzed with MIBI-TOF, with which we characterized the immunological landscape of TB granulomas from human patients and the CM NHP model of TB (5, 17, 18). Prior studies identified the high expression of IDO1 and suggested its immunoregulatory role in anti-*Mtb* T cell responses (9, 10, 16, 19, 20). Therefore, we aimed to perform similar high-dimensional analyses of the composition and phenotype of TB granulomas from D-1MT-treated vs. control animals to elucidate the immunoregulatory mechanism of IDO1.

To achieve this, we first generated a 30-plex panel of metal-labeled antibodies cross-reactive with RM epitopes (Fig. 1 *B* and *C* and *SI Appendix*, Fig. S2). This panel included markers to phenotype major immune and nonimmune cell lineages, including lymphocytes, macrophages, granulocytes, stroma, and epithelium (Fig. 1 *B* and *C*). Fig. 1*D* demonstrates the hematoxylin and eosin–stained tissue and two MIBI-TOF overlays of major lineage markers for an example granuloma from one of the D-1MT-treated RMs. We also included markers to evaluate immune regulation (IDO1), cell activation (CD40, HLA-DR, pS6), proliferation (Ki67), cytotoxicity [Granzyme B (GrzB)], Th1 cytokine production (IFNγ), and epigenetic state (H3K37me3, H3K9Ac) (Fig. 1 *B* and *C*). Representative staining patterns of all the markers included in this study are shown in Fig. 1*C*.

**Fig. 2.**
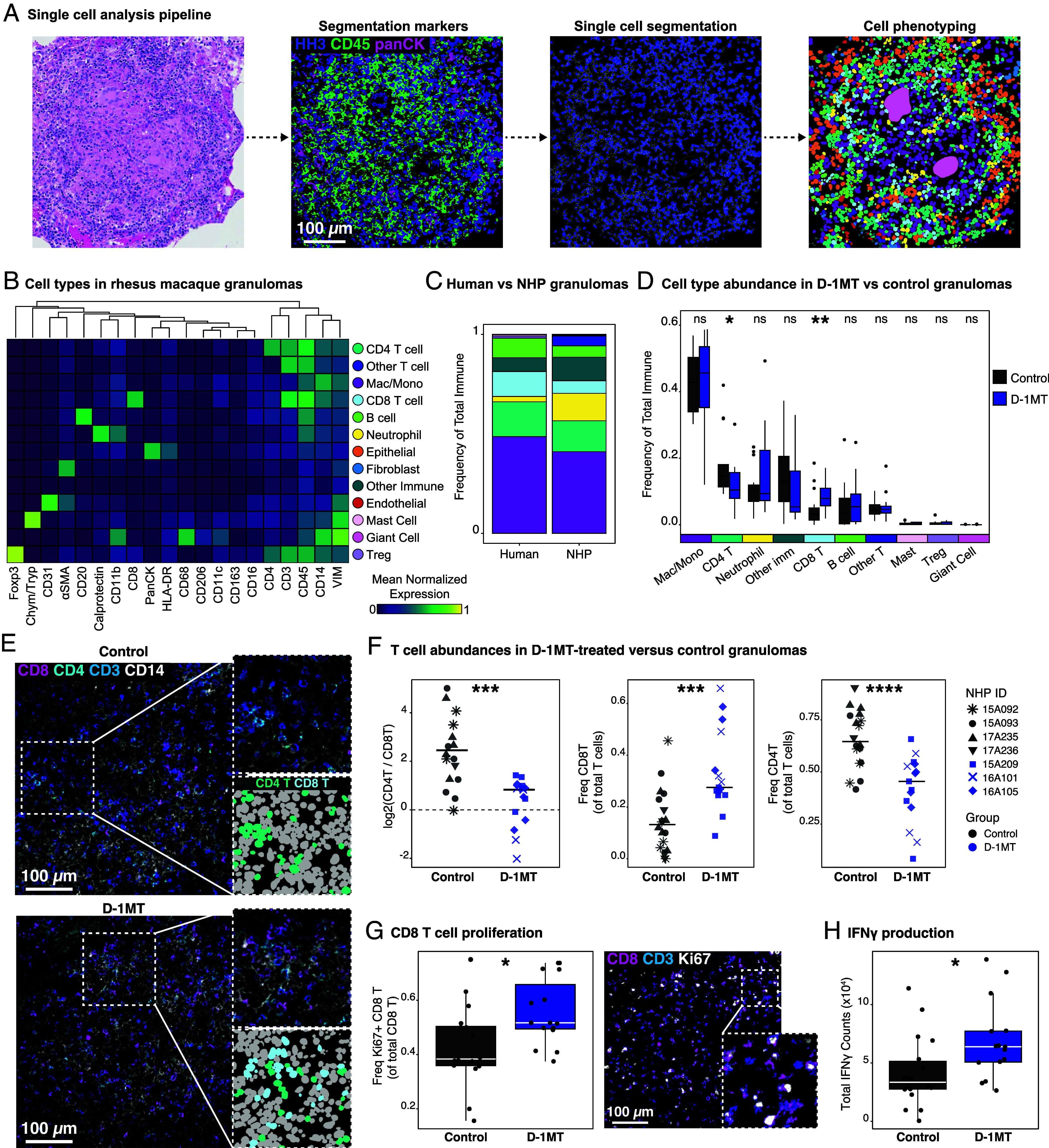
Cellular landscape of RM granulomas. (*A*) Graphical overview of the entire analytical pipeline applied to the dataset as part of this study, including cell segmentation and cell phenotyping. (*B*) Cell lineage assignments based on mean normalized expression of lineage markers (heatmap columns). Rows and columns are hierarchically clustered (Euclidean distance, average linkage). (*C*) Comparison of immune cell composition of *M. tuberculosis* granulomas from humans (from ref. 5) and from RMs. (*D*) Frequency distribution comparison of each immune cell subset between granulomas from D-1MT-treated RMs and control RMs. (*E*) MIBI-TOF expression overlays (*Top*—control RMs and *Bottom*—D-1MT-treatment RMs) demonstrating T cell subsets: CD8 (magenta), CD4 (cyan), CD3 (blue), and CD14 (white). Smaller *Insets* emphasize expression patterns (*Top*) and illustrate the presence of CD4^+^ T cells and CD8^+^ T cells in the cellular phenotype map (CPM): CD4^+^ T cells in teal and CD8^+^ T cells in light blue. (*F*) Comparison of the CD8^+^ T (*Center*) and CD4^+^ T (*Right*) cell frequencies of total T cells between control and D-1MT-treated RMs, on a per-granulomatous FOV basis. (*Left*) Comparison of the log2 ratio of CD4^+^ T and CD8^+^ T frequency between control and D-1MT-treated RMs. (*G*) (*Left*) Frequency of Ki67^+^ CD8^+^ T cells among control and D1-MT-treated RMs. (*Right*) Representative image of Ki67 (white), CD8 (magenta), and CD3 (blue) in the D-1MT-treatment granuloma shown in *E*. (*H*) Total IFNγ counts within granulomatous FOVs, compared between control and D-1MT-treated granulomatous lesions. (Scale bar: 100 μm.) All *P*-values were calculated with a Wilcoxon Rank Sum test (*P < 0.05, **P < 0.01, ***P < 0.001, ****P < 0.0001).

Following low-level processing of the imaging data (*Methods*), we performed single-cell segmentation with the deep-learning approach, Deepcell (21, 22) (Fig. 2*A*). Segmented cells were then partitioned into nonimmune cells (CD45^−^ panCK^+^/CD31^+^/aSMA^+^) or immune cells (CD45^+^ CD20^+^/CD14^+^/CD3^+^/Calprotectin^+^). Using iterative clustering, nonimmune cells were further resolved into fibroblasts, endothelial cells, and epithelial cells, and immune cells were clustered into one of nine subsets (lymphoid: CD4^+^, CD8^+^, regulatory T cells (Tregs), other T cells, and B cells; myeloid: macrophages/monocytes, neutrophils, and giant cells; and indeterminate: other immune lineage cells) (Fig. 2*B*). This allowed us to map the single-cell composition of each granuloma with our dataset comprising 68,296 cells across the 33 FOVs analyzed (*SI Appendix*, Fig. S3*A*). A similar study of human TB granulomas previously identified 20 different cell types using 24 different markers (5). Of these, we find that CD4^+^, CD8^+^ T cells, B cells, neutrophils, and giant cells were identified in both human and RM granulomas among immune cells while all three stromal cell types (epithelial, endothelial, fibroblasts) were commonly identified (Fig. 2*C* and *SI Appendix*, Fig. S3*B*). In conclusion, we demonstrate the successful application of the MIBI-TOF platform to RM tissues and bolsters the translational relevance of the NHP TB model for studying human TB disease.

### Comparison of Cellular Composition and State in D-1MT-Treated vs. Control TB Granulomas.

We next sought to compare the cellular composition of TB granulomas from D-1MT treated vs. control RMs by analyzing both the count and frequency of all cell subsets in each group (Fig. 2*D* and *SI Appendix*, Fig. S3 *C* and *D*). Of all the subsets identified, we found that only CD8^+^ T cells and CD4^+^ T cells varied between groups with a significant increase CD8^+^ T cell frequency (of total immune cells, *P* = 0.0036) and count (*P* = 0.0045) in the D-1MT-treated animals (Fig. 2*D* and *SI Appendix*, Fig. S3 *E* and *F*). Taking a closer look at the lymphocyte compartment of the granulomas, we found that this increase in CD8^+^ T cell frequency (of total T cells, *P* = 0.00063) coincided with a decrease the proportion of CD4^+^ T cells (*P* = 9.2e-5), leading to granulomas that were more CD8^+^ T cell–skewed (Fig. 2 *E* and *F*). In our prior study of these animals, we previously observed increases in both CD4^+^ and CD8^+^ T cells in the lung and bronchoalveolar lavage (BAL) fluid of D-1MT-treated RMs, yet, in the granuloma, it was only the CD8^+^ T cells that were elevated in the D-1MT-treated RMs. In that prior study, we had been unable to phenotype CD3^+^ T cells into either CD4^+^ or CD8^+^ T cell subsets by immunohistochemical confocal microscopy. Here, the use of MIBI-TOF clearly allowed us to identify these as CD8^+^ T cells. By evaluating historical controls, we also validated this increase in CD8^+^ T cells was not driven by infection time, given the later necropsy date of D-1MT infected animals (*SI Appendix*, Fig. S3*G*). This is a notable deviation from the CD4^+^ T cell–dominated response typically associated with anti-*Mtb* immunity in the granuloma.

We next asked whether this shift in the T cell composition of granulomas from D-1MT-treated RMs corresponded with changes in cell state or function (*SI Appendix*, Fig. S4 *A* and *B*). While a reduction in IDO1 enzymatic activity was previously reported by us in these animals (as indicated by the tryptophan: kynurenine ratio), we found only modest, yet significant, reduction in the expression of IDO1 across immune cells at the single-cell level (*P* = 0.00098, Fig. 2*I*) but not when summarized at the sample level (*SI Appendix*, Fig. S4 *C* and *D*). We also found no significant difference in the expression of CD40, HLA-DR, pS6, H3K9Ac, H3K27me3, or PD-L1 at either the single-cell or image level across cell subsets. Yet, we did observe that CD8^+^ T cells, but not CD4^+^ T cells, displayed elevated levels of Ki67, a marker of cellular proliferation (*P* = 0.02, Fig. 2*G* and *SI Appendix*, Fig. S4*E*), suggesting that increased CD8^+^ T cell abundance in the setting of D-1MT results from T cell expansion in the granuloma itself as opposed to increased recruitment. We found no difference in the proportion of CD8^+^ T cells that express GrzB between D-1MT-treated and control RMs (*SI Appendix*, Fig. S4*D*), indicating no difference in the cytotoxic capacity of CD8^+^ T cells with treatment. The same was true for all other subsets of GrzB^+^ cells (*SI Appendix*, Fig. S4 *G* and *H*). However, we did find increased quantities of IFNγ in granulomas from D-1MT-treated RMs relative to those from control RMs (*P* = 0.02, Fig. 2*H* and *SI Appendix*, Fig. S4 *I* and *J*).

D-1MT is a tryptophan mimetic and has been shown to restore mTOR activity via cellular accumulation of kynurenine (14, 15). Considering this, we sought to more deeply investigate how D-1MT treatment altered macrophage function in the context of mTOR signaling and activation. First, we found distinct subclusters of macrophages: Mac 1, Mac2, and Mac 3 (*SI Appendix*, Fig. S5*A*). We next evaluated expressions of CD40, Ki67, H3K9Ac, H3K27me3, HLA-DR, IFNγ, and pS6 in these subsets (*SI Appendix*, Fig. S5 *B* and *C*). Notably, we found that Mac 1, a subset with an inflammatory-like phenotype, frequency was elevated in granulomas from D-1MT-treated animals (*SI Appendix*, Fig. S5*D*, *P* = 0.04), while Mac 3, a monocyte-like population, had a decreased abundance in D-1MT treated animals (*SI Appendix*, Fig. S5*D*, *P* = 0.04). This suggests that a shift toward a more proinflammatory macrophage state is associated with D-1MT treatment.

With respect to mTOR activity we did not observe a difference in the mean expression of pS6 or frequency of pS6^+^ cells across any cell subset in D-1MT vs. control granulomas (*SI Appendix*, Fig. S5*E*). Notably, Mac 1, which was elevated in D-1MT-treated animals, expressed the highest levels of pS6, suggesting some association with its inflammatory phenotype (*SI Appendix*, Fig. S5 *B*–*D*). We also found that pS6 expression was elevated at the single-cell level in Ki67^+^ CD4^+^ and CD8^+^ T cells (*SI Appendix*, Fig. S5*F*), consistent with an activated phenotype. To more directly assess the relationship between the IDO1 and mTOR pathways, we performed in vitro treatment of bone marrow derived macrophages (BMDMs) with either D-1MT or the mTOR inhibitors, rapamycin and Torin1, and evaluated gene expression of *IDO1* and *MTOR* 24 and 48-h following *Mtb* infection (*SI Appendix*, Fig. S5 *G*–*I*). Consistent with our MIBI-TOF results, D-1MT-treated *Mtb*-infected BMDMs showed modest but significant downregulation of *IDO1* in a time-dependent (*P* = 0.0152) and dose-dependent (*P* = 0.0502) manner (*SI Appendix*, Fig. S5*G*). However, there was only a slight, but not significant, increase in *MTOR* expression. We also observed that rapamycin and Torin1 did not significantly affect the *MTOR* or *IDO1* transcript levels at 24 h or 48 h postinfection regardless of treatment dose (*SI Appendix*, Fig. S5 *H* and *I*). While this does not rule out that mTOR signaling is, in part, mediating the effects of D-1MT treatment, we did not observe strong evidence for its alteration in this setting. In conclusion, D-1MT treatment is associated with an expansion of CD8^+^ T cells in the granuloma, a switch to a more Th1-like cytokine environment, and an enrichment of proinflammatory macrophages.

### D-1MT Treatment Restructures the Spatial Organization of TB Granulomas.

T cell–macrophage interactions are crucial for mediating host immunity and improving mycobacterial killing. Canonically, the myeloid core is depleted of effector CD4^+^ and CD8^+^ T cells and instead is preferentially infiltrated by proliferating Tregs. In previous work using conventional fluorescence microscopy, we previously observed that granulomas from D-1MT-treated animals exhibited increased T cell infiltration, although we could not clearly identify these cells as either CD4^+^ or CD8^+^ T cells (23). Based on the observed elevation of CD8^+^ T cell frequency and proliferation, we hypothesized that D-1MT treatment could promote CD8^+^ T cells infiltration into the myeloid core.

**Fig. 3.**
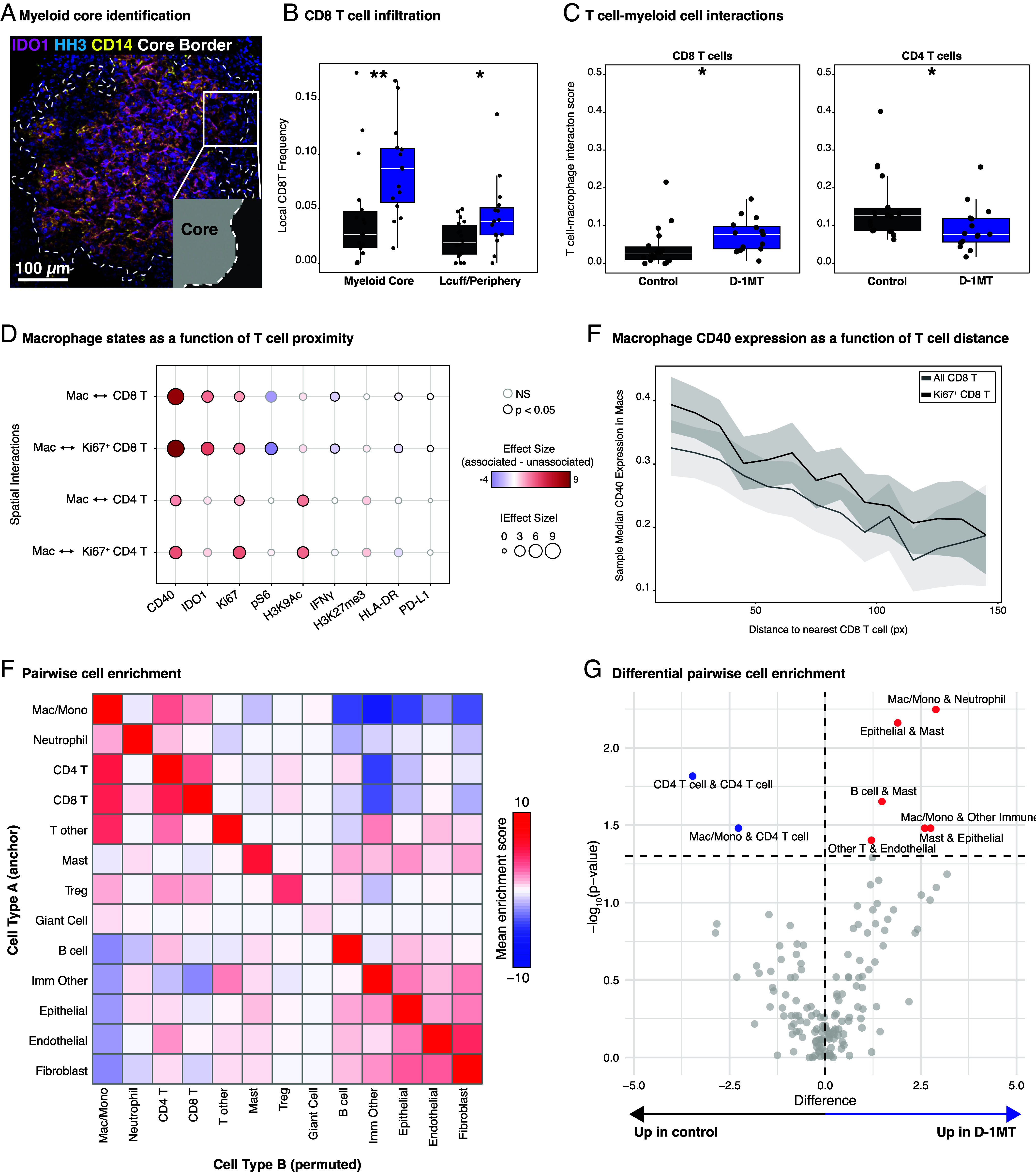
CD8^+^ T cell–macrophage/monocyte interactions are more prevalent in D-1MT-treated RM granulomas. (*A*) Representative granuloma with myeloid core border annotated (white, IDO1= magenta, CD14 = yellow, HH3 = blue) with zoomed *Inset* annotated the core (light gray) and periphery (dark gray). (*B*) Quantification of the local frequency of CD8^+^ T cells of total cells in the myeloid core or lymphocytic cuff (Lcuff)/periphery. (*C*) CD4^+^ T cell–myeloid cell (*Right*) and CD8^+^ T cell–myeloid cell (*Left*) mixing scores in control vs. D-1MT-treated granulomas. (*D*) Bubble chart summarizing the frequency of macrophages expressing the markers displayed along the columns when spatially interacting as shown along the rows. Bubble size and color represent the effect size. Statistically significant relationships (*P* < 0.05) are circled in black. *P*-values were calculated with a paired Wilcoxon Rank Sum test. (*E*) Median CD40 expression in macrophages with respect to distance from the nearest CD8^+^ T cell (gray) or Ki67^+^ CD8^+^ T cells (black). Solid lines represent the mean across 10-pixel bins and silhouettes represent the SE. (*F*) Global organization quantified by pairwise cell subtype enrichment (or depletion) in all granulomas with anchor subsets (cell type A) along the rows and permuted cell subsets (cell type B) along the columns. (*G*) The effect of D-1MT treatment of pairwise cell subtype enrichment shown as a volcano plot. *P*-values were calculated with a Wilcoxon Rank Sum test (*P < 0.05, **P < 0.01).

To test this, we automatically defined and masked the myeloid core region of each granuloma (Fig. 3*A*). Cells within these regions were assigned as “myeloid core-infiltrating,” while those outside the mask were assigned to the lymphocytic cuff (Lcuff)/periphery (*SI Appendix*, Fig. S6*A*). We found that CD8^+^ T cells were significantly enriched in the myeloid core of granulomas from D-1MT-treated RMs relative to control RMs (*P* = 0.0045, Fig. 3*B*). The CD8^+^ T cell frequencies were also significantly higher when measured in the Lcuff/peripheral region of granulomas, but to a lesser extent than in the myeloid core (*P* = 0.027, Fig. 3*B*).

We next assessed T cell–macrophage spatial associations to determine if D-1MT treatment increased cellular interactions between lymphocytes and myeloid cells. For quantification, we employed a mixing score that quantified the number of CD4^+^ or CD8^+^ T cells interactions with macrophages/monocyte relative to the number of homotypic macrophage/monocyte interaction (18, 24). This analysis revealed that the interactions between CD8^+^ T cells and monocyte-lineage cells were indeed significantly increased in D-1MT-treated RMs relative to control RMs (*P* = 0.011, Fig. 3*C*). Interestingly, granulomas from D-1MT-treated RMs had lower interaction scores between CD4^+^ T cells and macrophages/monocytes compared to those from control RMs (*P* = 0.018, Fig. 3*C*), suggesting that increased CD8^+^ T cell interactions may come at the expense of macrophage/monocyte-interactions with CD4^+^ T cells.

Next, we investigated how macrophage state is impacted by their increased interactions with CD8^+^ T cells. To do this, we quantified the frequency of macrophages positive for a variety of functional markers as a function of spatial association with CD4^+^ and CD8^+^ T cells, with or without expression of Ki67 (Fig. 3*D*). We found that expression of CD40 was markedly increased in macrophages spatially associated with CD8^+^ T cells (Fig. 3*D*, Ki67^+^ or Ki67^−^). To expand upon this, we also quantified the expression of CD40 in macrophages as a function of continuous distance from total CD8^+^ T cells and Ki67^+^ CD8^+^ T cells, confirming that CD40 is sharply upregulated by CD8^+^ T cell–associated macrophages (Fig. 3*E*).

Given the increased infiltration of the granuloma myeloid core by CD8^+^ T cells after D-1MT treatment, we next conducted pairwise cell enrichment to better understand the global spatial patterning of all cell subsets (5, 24). Consistent with the compartmentalization of macrophages in the myeloid core, we found that macrophages/monocytes were spatially enriched with themselves (Fig. 3*F*). Furthermore, T cells (CD4^+^ T cells, CD8^+^ T cells, and other T cells) displayed the strongest spatial enrichment with other T cells, consistent with their predominance in the lymphocytic cuff. When comparing granulomas from D-1MT-treated RMs with those from control RMs, we observed spatial enrichment of macrophages/monocytes with neutrophils and other immune cells as well as mast cells with B cells and epithelial cells in granulomas from D-1MT-treated animals (Fig. 3*G* and *SI Appendix*, Fig. S6*B*). Conversely, spatial enrichment of CD4^+^ T cells with other CD4^+^ T cells and with macrophages/monocytes were significantly reduced with D-1MT treatment (Fig. 3*G*). Taken together, D-1MT treatment is associated with spatial remodeling of the granuloma that favors increased interactions between CD8^+^ T cells and macrophages.

### Validation of Increased CD8^+^ T cell Infiltration into the Granulomas of D-1MT-Treated Animals by Confocal Microscopy.

**Fig. 4.**
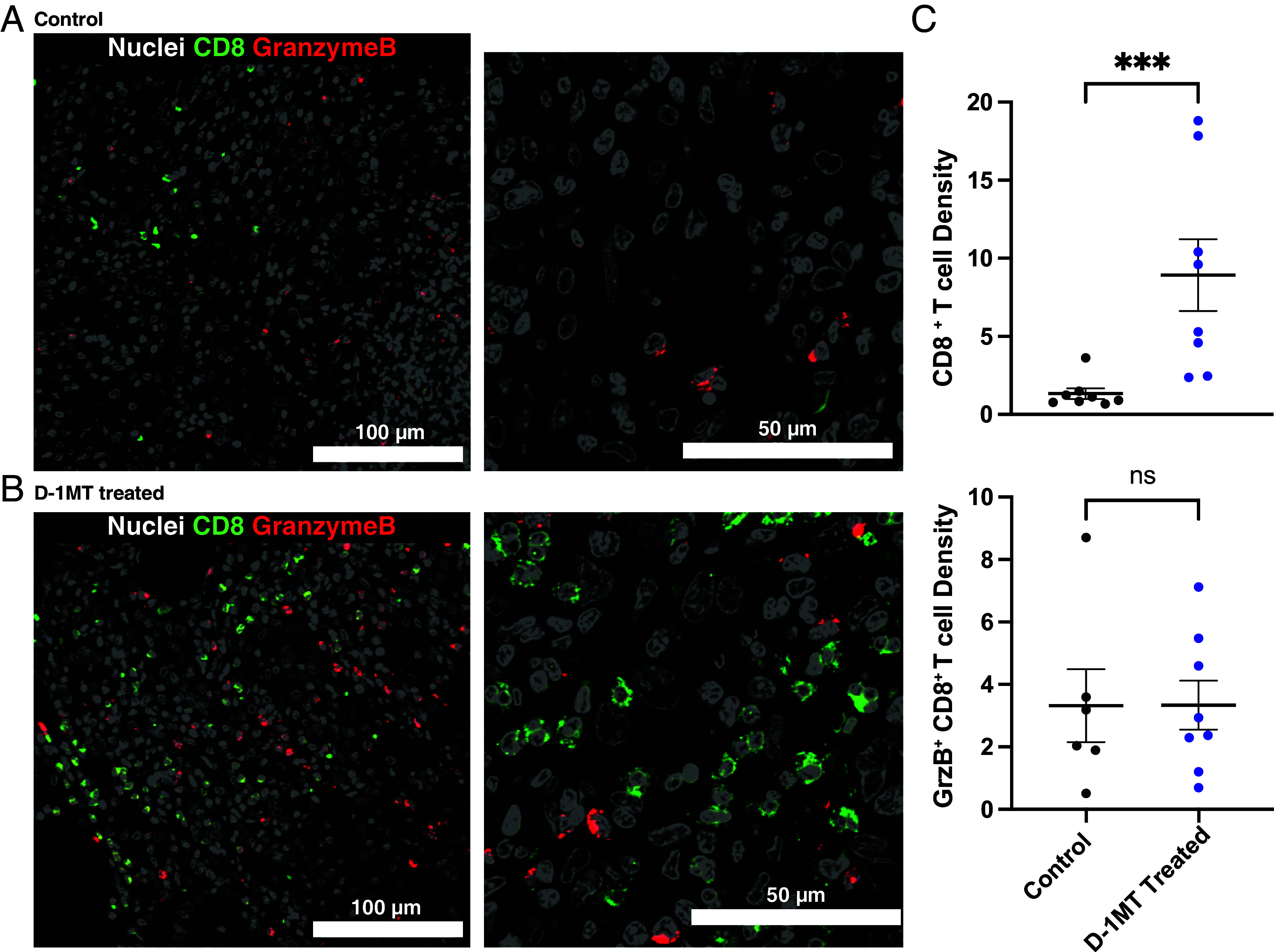
Confocal microscopy validates increased CD8^+^ T cell infiltration into the granulomas of D-1MT-treated RMs. CD8 (green), GrzB (red), and nuclear staining (white) in *Mtb*-infected (*A*) untreated RM granulomas (20× left; 63× right) and (*B*) D-1MT-treated RM granulomas (20× left; 63× right). (*C*) Frequency of CD8^+^ T cells (of all cells), stratified by granulomatous lesions from control RMs and D-1MT-treated RMs. (*D*) Percent of GrzB positivity among CD8^+^ T cells, stratified by granulomatous lesions from control RMs and D-1MT-treated RMs. All *P*-values were calculated with a Student’s *t* test (two tailed) (***P < 0.001).

To independently validate our MIBI-TOF findings, we stained FFPE sections from the same granulomas analyzed via MIBI-TOF with antibodies specific for CD8 and GrzB. We then imaged those samples with confocal microscopy (Fig. 4 *A* and *B*). Quantification of CD8^+^ T cell density across multiple sections revealed a significant increase in CD8^+^ T cells per unit area within granulomas from D-1MT-treated RMs (mean = 8.92%) compared to untreated controls (mean = 1.34%) RMs (*P* = 0.0006, Fig. 4*C*). Similarly, we observed that levels of GrzB expression (both on CD8^+^ T cells and on total CD3^+^ T cells) was comparable in treated vs. untreated animals (Fig. 4*C*). These results independently validate our high-dimensional imaging findings and support the conclusion that D-1MT treatment enhances CD8^+^ T cell infiltration into TB granulomas.

## Discussion

Granulomas are critical for the immune control of *Mtb* infection; however, not all granulomas effectively control TB disease. TB granulomas display heterogeneity in their primary function—*Mtb* killing capacity—as well as in cellular immune responses, during both the latent control of *Mtb* infection (LTBI) and progression to active TB disease (7, 25, 26). The basis of this functional heterogeneity is unclear. RMs are a robust, preclinical model for studying TB and can recapitulate features of both active TB and LTBI. Importantly, RM granulomas mirror the morphology and physiology observed in human disease (27). Immune correlates of TB disease in the lungs of RMs overlap significantly with those observed in the blood of human TB progressors (28). The accumulation of myeloid cells [e.g., plasmacytoid dendritic cells, pDCs, that express type I interferon (IFN); inflammatory, IDO1^+^ interstitial macrophages, IMs; and myeloid derived suppressor cells, MDSCs] is a dominant feature of granulomas during TB disease in both humans and RMs (16, 29–31). A protective role for lymphoid features, including increased inducible bronchus-associated lymphoid tissue and cytolytic effector-expressing NK cells, has also been defined within granulomas during *Mtb* control (29, 32, 33). The role of type I IFN instead is controversial, with some studies indicating a pathological effect and others suggesting a protective role (34–36). We have shown that D-1MT treatment downregulates expression of Type I IFN/numerous downstream Interferon Stimulated Genes (ISGs) correlating with lower IDO1 activity and improved infection control (10). Collectively, there are myriad cellular players and pathways influencing granuloma function during TB.

This study utilized MIBI-TOF on preserved lung granuloma sections from experimentally *Mtb*-infected RMs to better understand granuloma structure and function, including the role of IDO1 in suppressing anti-TB responses. We have recently used this innovative technique to spatially profile both human and CM TB granulomas at a single-cell resolution (5, 18). Granulomas from human TB patients were characterized by an intense immunosuppressive phenotype with high IDO1 and PD-L1 expresssions (5, 18). In the current study, we found that most immune and stromal cell populations present in human TB granulomas are also detected in RM TB granulomas. We also determined that immune cells make up at least 75% of all the cells in the RM granulomas. Approximately half of all the immune cells detected in RM TB granulomas are macrophages or monocytes. Besides neutrophils, which make up about 10% of immune cells, other myeloid cell populations, such as mast cells and giant cells, were detected at much lower frequencies. Among lymphocytes, CD4^+^ T cells were the most frequent followed by B cells, CD8^+^ T cells, other (likely γδ T cells) T cells, and Tregs, in order of decreasing frequency.

The finding that CD8^+^ T cell frequency is significantly increased in granulomas, specifically in the myeloid core, of D-1MT-treated macaques is of interest. Furthermore, this finding clarifies a conundrum from our prior studies (10). We previously identified that D-1MT treatment led to greater T cell access to the granuloma core (10); however, we were unable to phenotype these T cells as either CD4^+^ or CD8^+^ T cells by microscopy. Both CD4^+^ and CD8^+^ T cells exhibited increased proliferative capacity after D-1MT treatment in the BAL (10). Taken together with the significantly greater body of work suggesting the greater importance of CD4^+^ over CD8^+^ T cells in the structure and function of the TB granuloma, we predicted that D-1MT treatment would enhance the trafficking of CD4^+^ T cells to the lesion core. Instead, our current results clearly suggest that CD8^+^ and not CD4^+^ T cells infiltrate the granuloma core in higher numbers during D-1MT treatment. These results are consistent with high levels of GrzB expression on the CD3^+^ T cells trafficking to the core in our prior study (10). The role of CD8^+^ T cells in controlling *Mtb* infection is not fully understood. While CD8^+^ T cells were earlier thought to be less important that CD4^+^ T cells in the immune control of *Mtb*, their role is now being increasingly recognized as necessary yet complex (37). Mice lacking the ability to generate functional CD8^+^ T cells or those where these cells were depleted have an impaired ability to control *Mtb* (38–40). CD8^+^ T cells recruited in response to *Mtb* infection are antigen-specific, exhibit memory phenotype, and impart protection independent from CD4^+^ T cells during vaccination (41–44). A role for CD8^+^ T cells in anti-TB immunity in macaques is known (45). According to our results, activated CD8^+^ T cells gain access to the granuloma core upon reduction of IDO1 activity. These CD8^+^ T cells are highly proliferative (Ki67^+^), associate with a Th1 cytokine shift (increased IFNγ and decreased IDO1), express cytolytic effectors (GrzB), and correspond with activated and inflammatory macrophages. Thus, inhibition of IDO1 via D-1MT remodels the TB granuloma, providing activated, proliferative, cytokine producing CD8^+^ T cells access to the lesion core in the vicinity of infected myeloid cells.

These results are supported by the evidence that overexpression of IDO1 attenuates the generation of central memory and effector CD8^+^ T cells, while suppressing IDO1 activity promotes their generation (46), accompanied by defects in production of granule cytotoxic proteins, perforin, and Granzyme A/B (47). Furthermore, CD8^+^, and not CD4^+^, T cells dominate the immunosuppressed milieu in response to mycobacterial infection (48). We hypothesize that the greater interaction between these CD8^+^ T cells and infected myeloid cells leads to enhanced *Mtb* killing, resulting in better control of infection. Several key questions remain from this study. First, we identified a significant proportion of T cells in the studied granulomas that were neither conventional CD4^+^ or CD8^+^ T cells, suggesting they may be unconventional lymphocytes, such as γδ T cells, NKT cells, or MAIT cells. Future analyses should probe the identity and contribution of these cells in the setting of D-1MT treatment. Additionally, our study was restricted to solid nonnecrotizing granuloma and, therefore, may not capture the complete spectrum of TB histopathology. Regardless, these results provide strong rationale to better understand the role, mechanistically, of CD8^+^ T cells in the killing of *Mtb* intragranulomatously.

## Methods

### NHP Samples, Antibody Preparation, and Tissue Staining for MIBI-TOF.

Rhesus macaques (RMs) were infected and treated as described by Gautam et al. (10). Fourteen FFPE blocks of pulmonary tissues from four control, *Mtb* CDC1551-infected and three *Mtb* CDC1551-infected/D-1MT treated RMs were used for MIBI-TOF imaging experiments. In total, 18 granulomas from controls and 15 from D-1MT-treated RMs were analyzed (Fig. 1*A*). 5 μm serial sections of each specimen were stained with hematoxylin and eosin and two-six 500 μm^2^ FOVs were selected from each block for imaging via MIBI-TOF. To accurately capture granuloma microanatomy in these regions, we prioritized imaging FOVs with smaller, nonnecrotizing cellular granulomas. One RM lymph node and one RM spleen specimen were included as technical controls. Antibodies were conjugated to isotopic metal reporters, diluted, stored, reconstituted, and used as described previously (5). Information on the antibodies and metal reporters used for NHP experiments and staining concentrations is in *SI Appendix*, Table S1.

### Interactive Protocols.

Reagent preparation: https://doi.org/10.17504/protocols.io.bhmej43e

IHC staining: https://doi.org/10.17504/protocols.io.bf6ajrae

MIBI-TOF staining: https://doi.org/10.17504/protocols.io.dm6gprk2dvzp/v5

### MIBI-TOF Imaging.

Imaging was performed using a MIBI-TOF instrument with a Hyperion ion source as previously described (17). All samples were stained with a 30-plex antibody panel (*SI Appendix*, Table S1 and Fig. 1 *B* and *C*) and imaged with the MIBI-TOF platform. Multiplexed image sets were extracted, slide background-subtracted, denoised, and aggregate filtered using a custom low-level processing pipeline described by us previously (49). In addition to these processing steps, image compensation was performed to account for signal spillover due to adducts and oxides for the following interferences: Collagen-1 to IDO1 and Lag3, H3K9Ac to panCK and MPO, Chym/Tryp to MPO, Ki67 to CD209, CD20 to CD16, CD16 to IFNγ, CD11c to IDO1, and HLA-DR-DQ-DP to CD11b.

### Cell Segmentation and Phenotyping.

Nuclear segmentation was performed using an adapted version of Deepcell, a convolutional neural network that can be trained to predict single-cell segmentation across a range of biological platforms (21, 22). This algorithm was trained on 2,600 images of cells of diverse shapes and morphologies from nine different tissue types. Rather than predicting the nucleus and performing a radial expansion, this algorithm directly predicts the shape of the entire cell. The updated algorithm was used to generate segmentation predictions for each cell in the image with HH3 and CD45/pan-Keratin as nuclear and cell membrane channels, respectively, as input. Multinucleated giant cells were manually segmented in ImageJ and merged with the Deepcell-generated segmentation masks. Single-cell data were extracted and normalized as described above. Single-cell data were extracted for all cell objects and area-normalized. Cells with a sum of less than 0.1 area-normalized counts across all lineage channels were excluded from analysis. Single-cell data were linearly scaled with a scaling factor of 100 and asinh-transformed with a cofactor of 5. All mass channels were scaled to 99.9th percentile. To assign each cell to a lineage, the FlowSOM clustering algorithm was used with the Bioconductor “FlowSOM” package in R. FlowSOM clustering was applied to assign each segmented cell to one of thirteen phenotypes (Fig. 1*B*), and the proportion of cell types was quantified per granuloma FOV. Marker thresholds for each channel were automatically assigned using the MetaCyto silhouette scanning approach (50).

### Spatial Statistics.

To compare spatial features between groups, the IDO1 channel was used to produce a mask of the myeloid core as its expression appeared highly compartmentalized to regions expressing CD11c, CD11b, and CD14. The mask was produced by first capping (cap = 10 counts), Gaussian-blurring (sigma = 5), and binarizing the IDO1 channel. Next, close objects in the mask were connected using Matlab’s “imclose” function and objects with a size less than 10,000 pixels were filtered out of the mask. The mask was further smoothed by filling in holes with the “imfill” function, dilating the mask, and applying active contouring. Anything within the mask boundary was considered part of the myeloid core, while anything outside the mask was annotated as part of the “periphery.” The local proportion of CD8^+^ T cells was quantified in both zones relative to the total number of cells in each zone. To further assess the interaction between lymphocytes and myeloid cells, a mixing-score was calculated to quantify the degree of interaction between CD4^+^ and CD8^+^ T cells with macrophages and monocytes. The score was adapted from a tumor-immune mixing score presented by ref. 24 and was calculated as (total # CD4^+^ or CD8^+^ T cell–myelomonocytic interactions/total # myelomonocytic–myelomonocytic interactions), where an interaction was defined as two cells with <10 μm centroid–centroid distance (24). The higher the score, the higher the degree of T cell–myelomonocytic cell interactions.

To evaluate how macrophage phenotype varies as a function of spatial proximity with T cells, we performed two analyses. First, we annotated all macrophages as being spatially associated with either all CD4^+^ T cells, all CD8^+^ T cells, Ki67^+^ CD4^+^ T cells, or Ki67^+^ CD8^+^ T cells if they were within 20 pixels centroid–centroid distance of each other. Next, we determined the frequency of macrophages positive for expression of CD40, IDO1, Ki67, pS6, H3K9Ac, IFNγ, H3K27me3, and PD-L1 per sample and per group (spatially associated or not). To summarize these data, we determined the effect size per marker and spatial association by subtracting the median frequency of marker-positive macrophage in spatially associated vs. unassociated macrophages. To further quantify the relationship between CD40 expression of macrophages and CD8^+^ T cells, we plotted the median expression of CD40 on macrophages as a function of continuous distance from all CD8^+^ T cells or Ki67^+^ CD8^+^ T cells. To achieve this, the data were binned in 10-pixel intervals, then at each interval a curve was plotted of the mean and SE across bins.

To characterize how cellular niches organize at a global level in the granuloma, pairwise enrichment analysis was applied (adapted from ref. 24). For each cell, the physical distance to all other cells in the FOV was calculated and stored as a distance matrix. For each cell type A and B, interactions within 100 pixels (~50 μm) were counted as close to account for larger multicellular niches. Bootstrapping was applied to evaluate whether the number of close interactions is significant compared to interactions when the location of cell type B was randomized. This process was repeated 1,000 times to generate a null distribution, and a z-score was calculated to assess the deviation of the actual number from the null distribution. The z-scores were averaged across all FOVs to generate a heatmap of the pairwise enrichment, with the rows hierarchically clustered. The z-scores per FOV for each cell–cell interactions were sorted into control and D-1MT groups, with which Wilcox hypothesis testing was performed, and the log2 fold change was calculated.

### In Vitro BMDM Experiments.

Bone marrow was obtained from the opportunistic naïve necropsies of RMs. The isolated bone marrow cells were differentiated into BMDMs by treating them with GM-CSF (10 ng/mL) and replenishing half of the media (IMDM+10% FBS) every second day through day 7. Approximately 3 million BMDMs were seeded and treated with mTOR inhibitors (rapamycin and torin1) or IDO1 inhibitor (D-1MT) or vehicle (DMSO) at different concentrations for 2 h followed by infection with *Mtb* CDC1551 at MOI of 4 for 3 to 4 h for bacterial uptake. Then cells were washed and treated with 200 μg/mL Amikacin and incubated for 1 h. Fresh medium was added with inhibitors and incubated for 24 and 48 h. After incubation, cells were harvested in TRIzol reagent and RNA was isolated using Direct-zol RNA Miniprep kit (Zymo Research) and quantified with Qubit 4 Fluorometer using Qubit RNA BR assay kit (ThermoFisher Scientific). RNA was then reverse-transcribed to cDNA using dNTPs, random primers, RNase inhibitor, and Superscript Reverse Transcriptase (Invitrogen). RT-qPCR was performed in Quantstudio 6 Real-time PCR system using gene specific TaqMan Gene Expression Assays for RM. The assay Ids used were Rh02841203_m1 (*IDO1*) and Rh01042404_m1 (*MTOR*). The expression of target genes was normalized to *ACTB* gene and fold change was calculated by ΔΔCt method relative to *Mtb*-infected samples without inhibitors.

### Confocal Microscopy.

To validate the findings of lung granuloma MIBI-TOF imaging, multilabel immunohistochemistry was performed on *Mtb*-infected RM lungs with active TB with and without D-1MT treatment. The lung sections were stained with anti-CD8 (Polyclonal, Cat no: HPA037756, Sigma-Aldrich) and anti-Granzyme-B (Clone-GrB-7, Cat no: M7235, Dako) antibodies. DAPI was used for nuclear staining. Images were captured using a Zeiss LSM-800 confocal microscope at 20× and 63× magnification. For quantification, the slides were scanned on Zeiss Axio Scan Z1, and CD8^+^ T cells as well as GrzB expression in the granuloma regions of the lung were quantified using HALO software (Indica Labs).

### Software.

Image processing was conducted with Matlab 2016a and Matlab 2019b. Statistical analysis was conducted in Matlab 2016a, Matlab 2019b, and R version 3.6.2. Data visualization and plots were generated in R. Representative images were processed in Adobe Photoshop and figures were prepared in Adobe Illustrator. Schematic visualizations were produced with Biorender.

## Supplementary Material

Appendix 01 (PDF)

## Data Availability

All original code utilized in this study can be accessed at https://github.com/angelolab/publications/tree/main/2025-McCaffrey-Delmastro_etal_D1MT (51). The MIBI-TOF dataset analyzed here is available through Mendeley’s data repository at DOI: 10.17632/x5sf8gpr67.1 (52). All other data are included in the manuscript and/or *SI Appendix*.
