# Supplementary material for: Therapeutic remodeling of the tuberculosis granuloma with 1-methyl-D-tryptophan enhances CD8+ T cell–macrophage interactions
Source: Proc Natl Acad Sci U S A. 2026 Aug 11;123(33):e2528104123. doi: 10.1073/pnas.2528104123 (PMC13486567; doi:10.1073/pnas.2528104123)
Supplement: Supplementary file 1 [file pnas.2528104123.sapp.pdf]

Figure S1

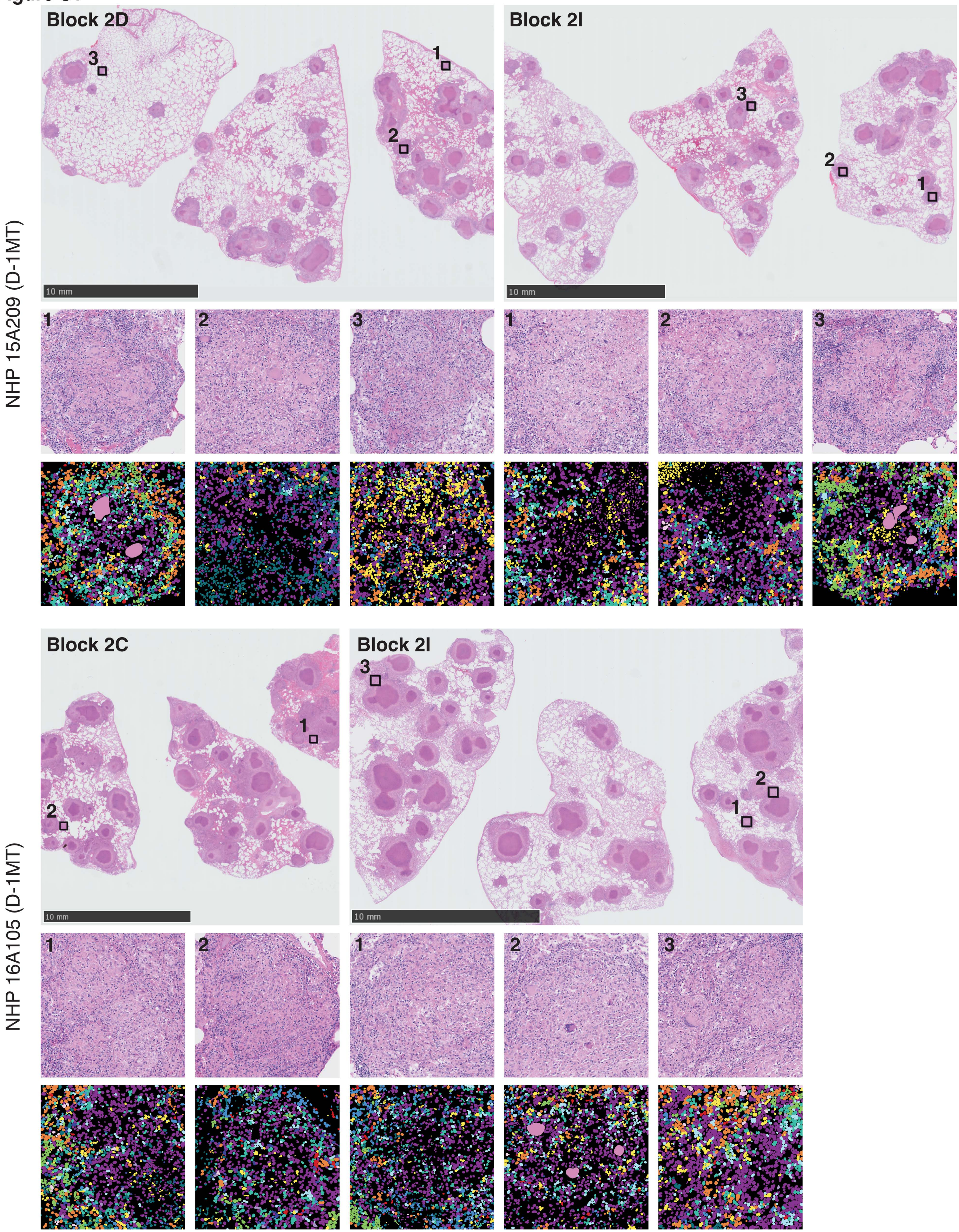
Block 2D
Block 2I
10 mm
10 mm
NHP 15A209 (D-1MT)
1
2
3
1
2
3
Block 2C
Block 2I
10 mm
10 mm
NHP 16A105 (D-1MT)
1
2
1
2
3

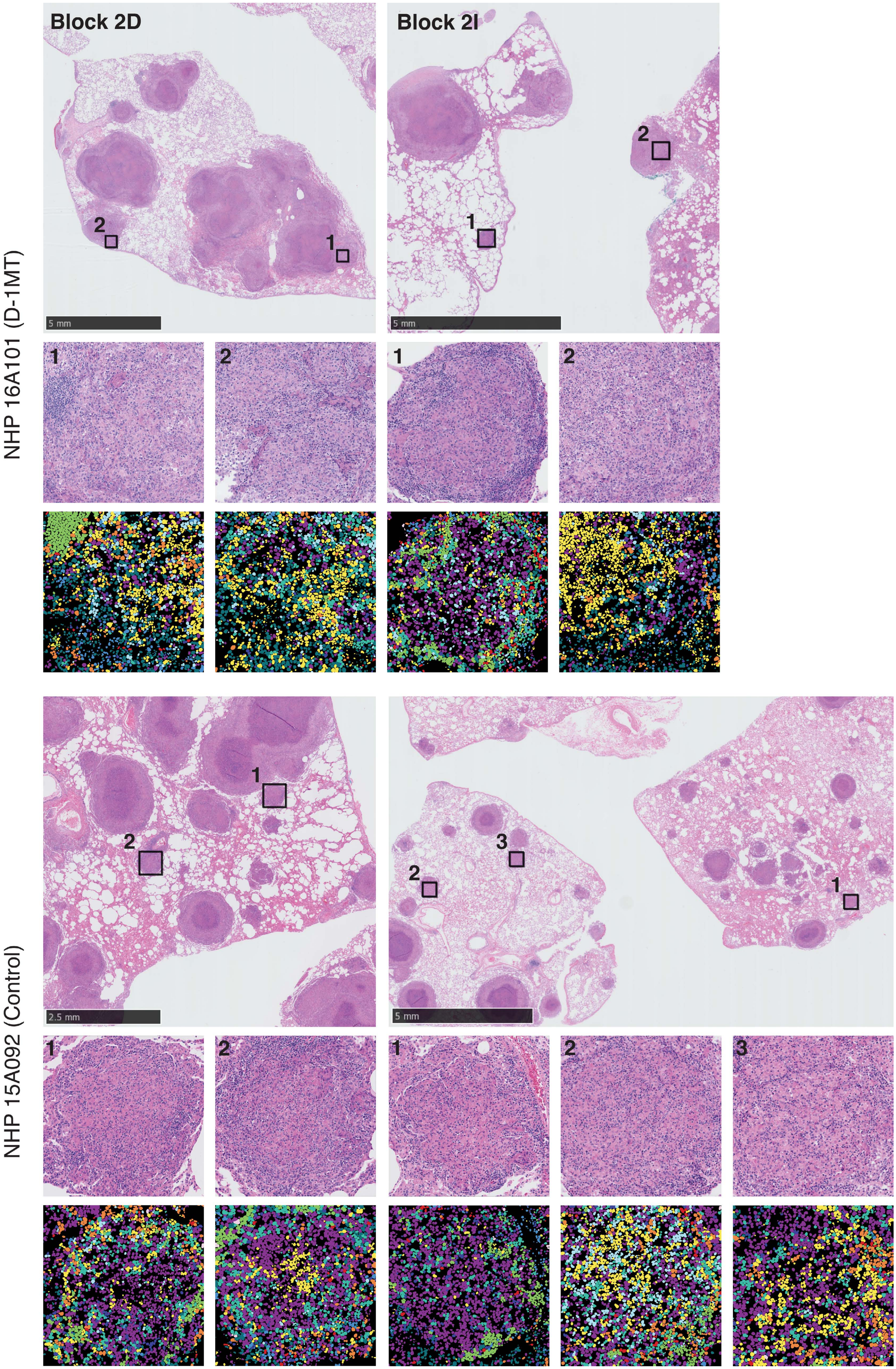

Block 2D
Block 2I
NHP 16A101 (D-1MT)
NHP 15A092 (Control)
5 mm
5 mm
2.5 mm
5 mm
1
2
1
2
1
2
3

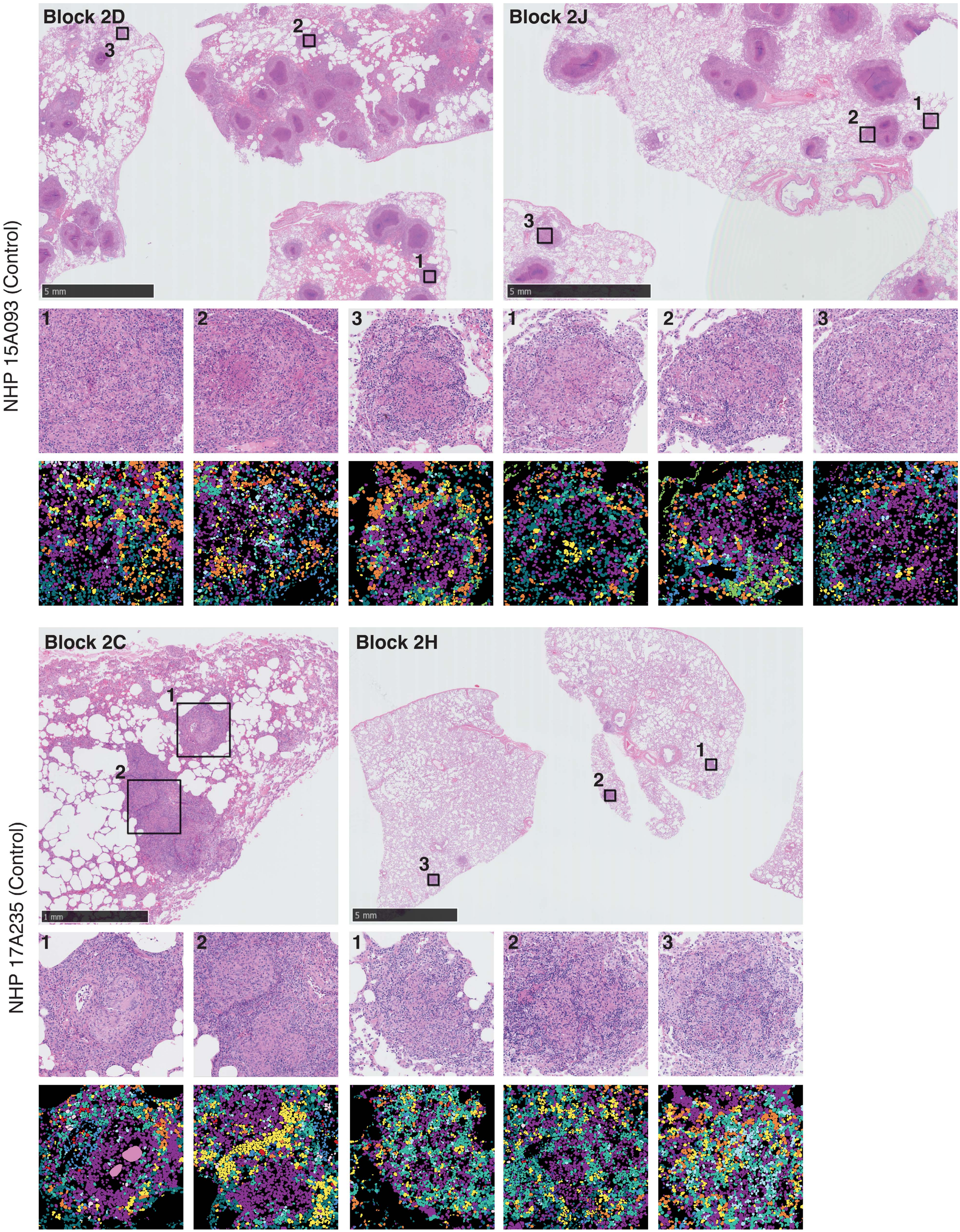
NHP 15A093 (Control)
Block 2D
Block 2J
5 mm
5 mm
NHP 17A235 (Control)
Block 2C
Block 2H
1 mm
5 mm

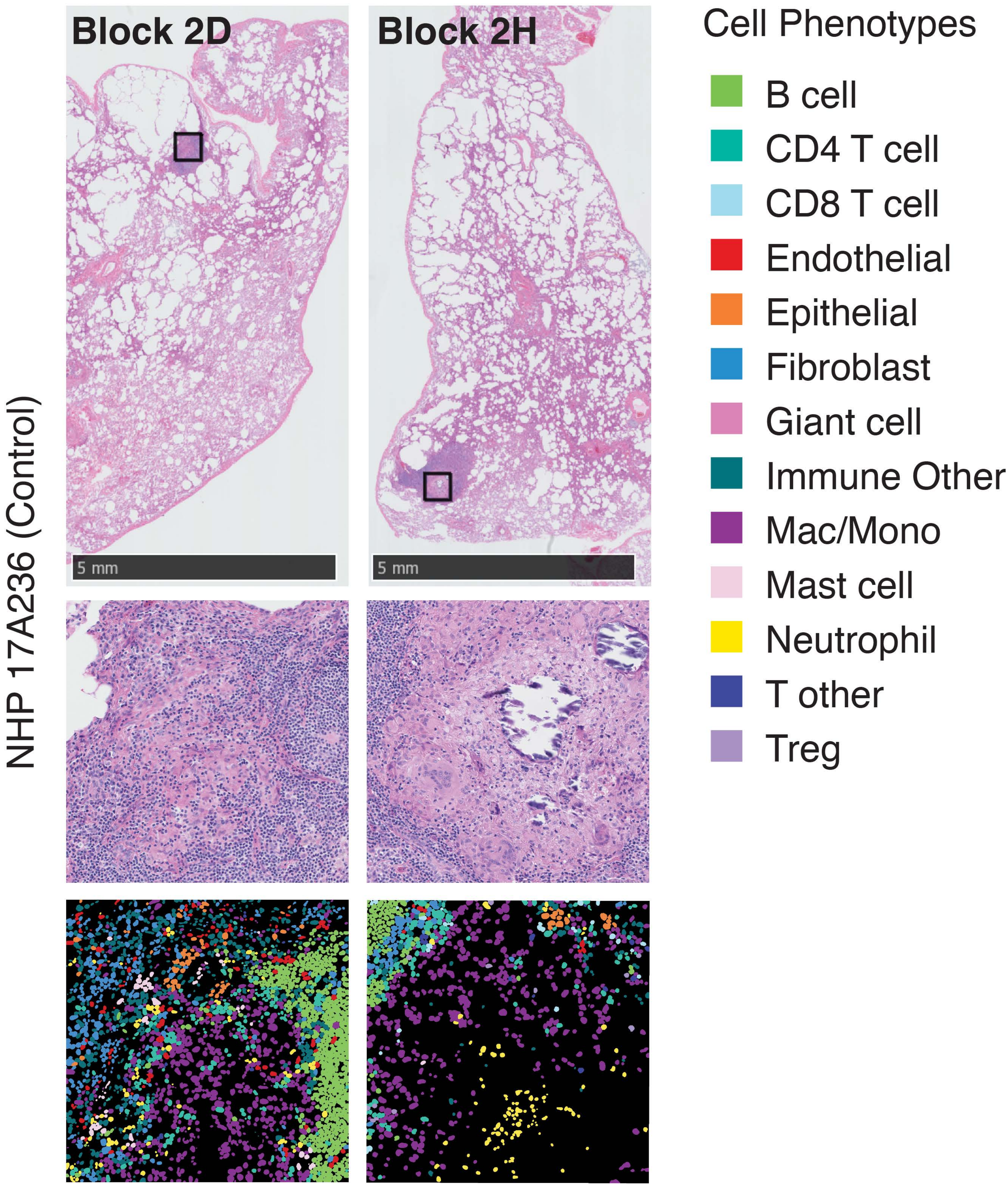
NHP 17A236 (Control)
Block 2D
Block 2H
5 mm
5 mm
Cell Phenotypes
B cell
CD4 T cell
CD8 T cell
Endothelial
Epithelial
Fibroblast
Giant cell
Immune Other
Mac/Mono
Mast cell
Neutrophil
T other
Treg

Figure S1: FOV selection and acquisition for each FFPE specimen by rhesus macaque. For each non-human primate (NHP), the first row demonstrates the hematoxylin and eosin-stain of the FFPE specimen, which the locations of each FOV. The second row includes the insets for each 500 x 500 μm^2 FOV acquired. The third row has the cell phenotype maps (CPMs) for each FOV acquired with MIBI-TOF.

Figure S2

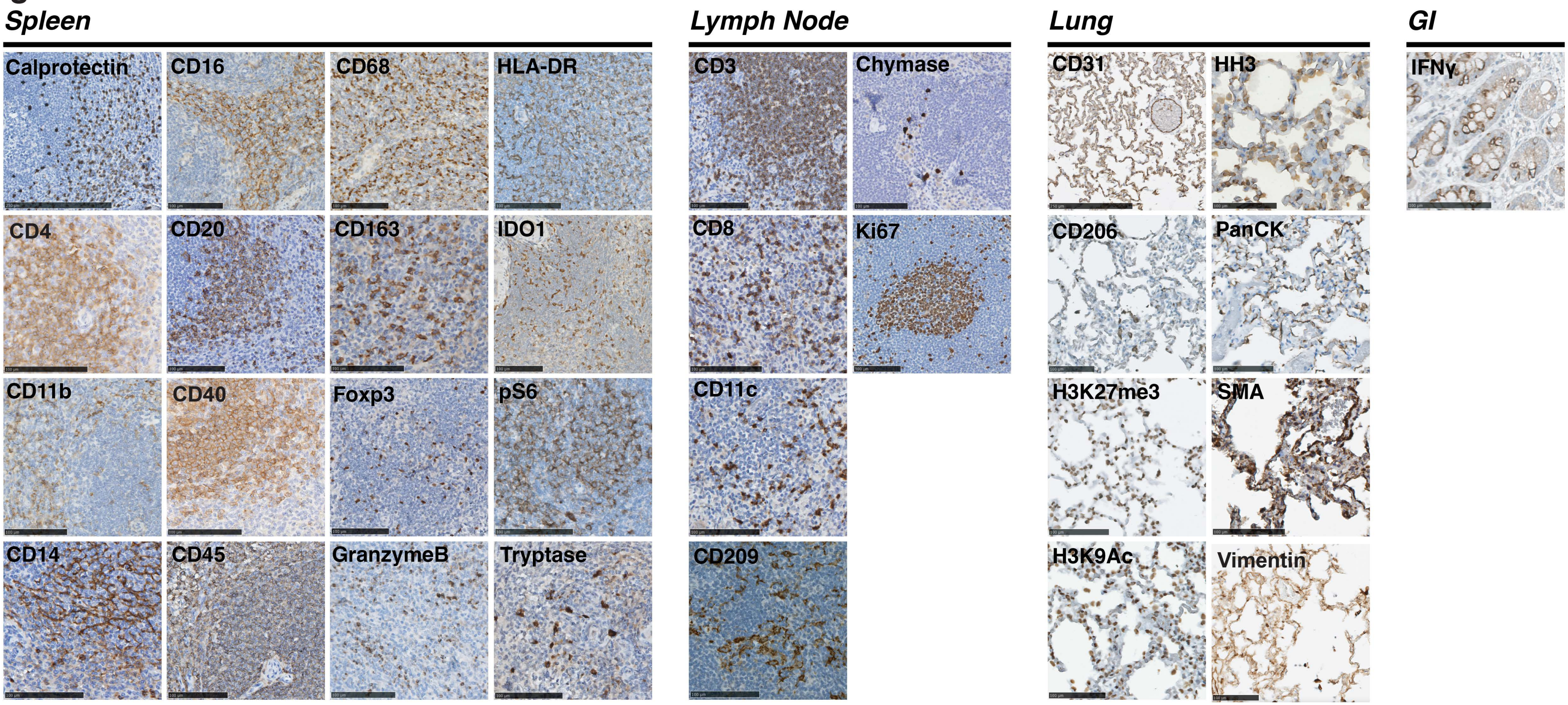
Spleen
Calprotectin
CD16
CD68
HLA-DR
CD4
CD20
CD163
IDO1
CD11b
CD40
Foxp3
pS6
CD14
CD45
GranzymeB
Tryptase
Lymph Node
CD3
Chymase
CD8
Ki67
CD11c
CD209
Lung
CD31
HH3
CD206
PanCK
H3K27me3
SMA
H3K9Ac
Vimentin
GI
IFNγ

Figure S2: Validation of rhesus macaque-reactive antibody reagents. Representative chromogenic immunohistochemistry images for all antibody targets in spleen, lymph node, lung, or gastrointestinal (GI) tissue.

Figure S3

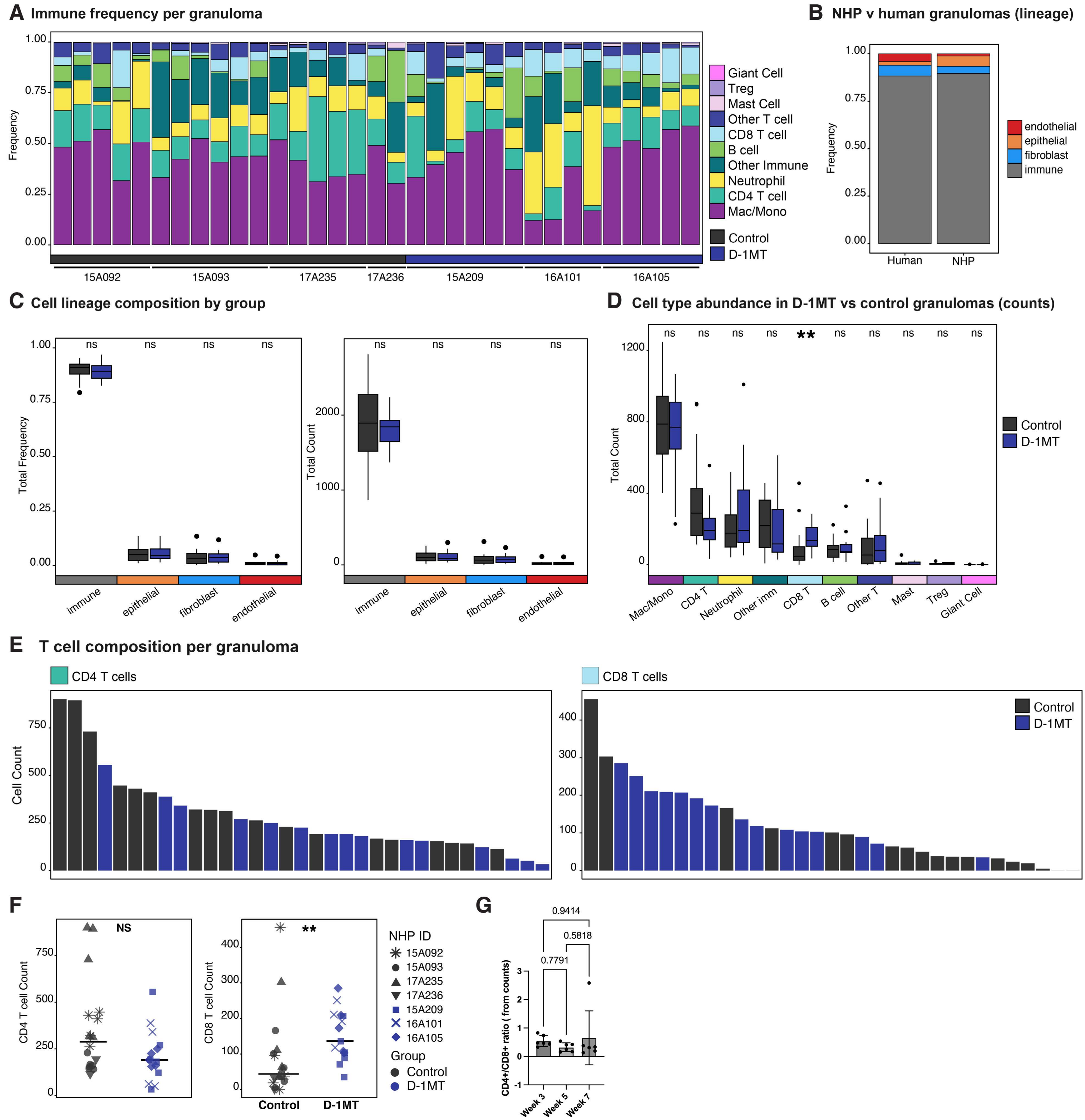

A Immune frequency per granuloma
Frequency
1.00
0.75
0.50
0.25
0.00
Giant Cell
Treg
Mast Cell
Other T cell
CD8 T cell
B cell
Other Immune
Neutrophil
CD4 T cell
Mac/Mono
Control
D-1MT
15A092
15A093
17A235
17A236
15A209
16A101
16A105
B NHP v human granulomas (lineage)
Frequency
endothelial
epithelial
fibroblast
immune
Human
NHP
C Cell lineage composition by group
Total Frequency
Total Count
ns
immune
epithelial
fibroblast
endothelial
D Cell type abundance in D-1MT vs control granulomas (counts)
Total Count
ns
**
Control
D-1MT
Mac/Mono
CD4 T
Neutrophil
Other imm
CD8 T
B cell
Other T
Mast
Treg
Giant Cell
E T cell composition per granuloma
CD4 T cells
CD8 T cells
Cell Count
Control
D-1MT
F
CD4 T cell Count
NS
CD8 T cell Count
**
Control
D-1MT
NHP ID
15A092
15A093
17A235
17A236
15A209
16A101
16A105
Group
Control
D-1MT
G
CD4+/CD8+ ratio (from counts)
0.9414
0.5818
0.7791
Week 3
Week 5
Week 7

Figure S3: Cellular composition of study samples. (A) Proportion of each immune cell subset (of all immune cells) per acquired FOV and categorized by RM and treatment group. Cell subsets are ordered by median frequency. **(B)** Proportion of major lineages (endothelial, epithelial, fibroblast, and immune) across of RM FOVs in comparison to those from human samples (McCaffrey et. al., 2022). Major lineages are order alphabetically, in descending order. **(C)** Frequency (left) and total counts (right) distribution comparison of each major lineage between granulomas between control and D-1MT-treated RMs. **(D)** Total counts distribution comparison of each immune cell subset (of all immune cells) between granulomas from D-1MT-treated RMs and control RMs. **(E)** Total counts of CD4+ T cells (left) and CD8+ T cells (right) for all FOVs, colored by treatment group and ordered in descending order. **(F)** Quantification of CD4+ T cell (top) and CD8+ T cell (bottom) total counts, stratified by FOVs of granulomas from control RMs and D-1MT-treated RMs. **(G)** The CD4+/CD8+ T cell ratio of historical controls of RMs at 3, 5, or 7-weeks post-infection with p-values calculated with a one-way ANOVA test. Unless noted, all p-values were calculated with a Wilcoxon Rank Sum test (ns $p > 0.05$, $^{**}p < 0.01$).

Figure S4

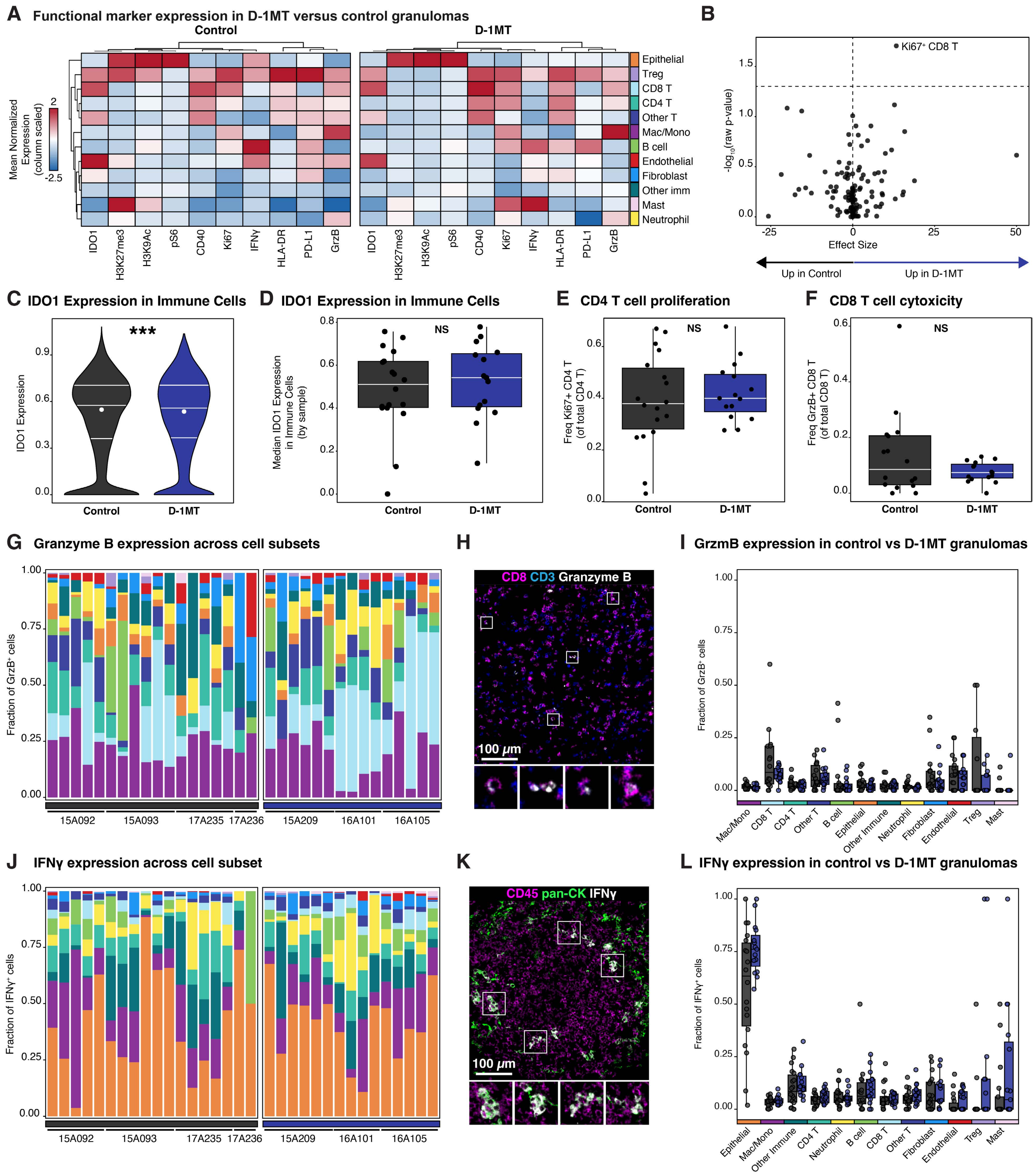

A Functional marker expression in D-1MT versus control granulomas
Control
D-1MT
Mean Normalized Expression (column scaled)
Epithelial
Treg
CD8 T
CD4 T
Other T
Mac/Mono
B cell
Endothelial
Fibroblast
Other imm
Mast
Neutrophil
IDO1
H3K27me3
H3K9Ac
pS6
CD40
Ki67
IFNγ
HLA-DR
PD-L1
GrzB
B
Ki67+ CD8 T
-log10(raw p-value)
Effect Size
Up in Control
Up in D-1MT
C IDO1 Expression in Immune Cells
IDO1 Expression
***
Control
D-1MT
D IDO1 Expression in Immune Cells
Median IDO1 Expression in Immune Cells (by sample)
NS
E CD4 T cell proliferation
Freq Ki67+ CD4 T (of total CD4 T)
F CD8 T cell cytoxicity
Freq GrzB+ CD8 T (of total CD8 T)
G Granzyme B expression across cell subsets
Fraction of GrzB+ cells
15A092
15A093
17A235
17A236
15A209
16A101
16A105
H
CD8 CD3 Granzyme B
100 μm
I GrzmB expression in control vs D-1MT granulomas
Fraction of GrzB+ cells
Mac/Mono
CD8 T
CD4 T
Other T
B cell
Epithelial
Other Immune
Neutrophil
Fibroblast
Endothelial
Treg
Mast
J IFNγ expression across cell subset
Fraction of IFNγ+ cells
K
CD45 pan-CK IFNγ
100 μm
L IFNγ expression in control vs D-1MT granulomas
Fraction of IFNγ+ cells

Figure S4: Immune cell functional status in RM granulomas. (A) Heatmap representation of functional marker expression (columns) across cell subsets (rows) in control (left) versus D-1MT-treated (right) RMs. Heatmaps display the mean normalized expression, scaled by column. **(B)** Volcano plot of the difference in frequency of marker-positive cell subsets in D-1MT-treated (right) versus control RMs (left). **(C)** Violin plots denoting IDO1 expression among total immune cells by treatment group. **(D)** Boxplots of the sample-level median IDO1 expression among total immune cells by treatment group. **(E)** Frequency of Ki67+ CD4+ T cells among control and D1-MT-treated RMs. **(F)** Frequency of Granzyme B (GrzB)+ CD8+ T cells among control and D1-MT-treated RMs. **(G)** Breakdown of Granzyme B+ cells by subset (of total Granzyme B+ cells) per acquired FOV and categorized by RM and treatment group. Cell subsets are ordered by median frequency. **(H)** Representative image of Granzyme B (white) in CD3+ (blue) CD8+ (magenta) T cells. **(I)** Frequency of Granzyme B+ cells (out of total cells) broken down by subset between granulomas from D-1MT-treated RMs and control RMs. Subset are ordered as in G. **(J)** Breakdown of IFNγ+ cells by subset (of total IFNγ+ cells) per acquired FOV and categorized by RM and treatment group. Cell subsets are ordered by median frequency. **(K)** Representative image of IFNγ+ (white) cells in CD45+ immune cells (magenta) and panCK+ (green) epithelial cells. **(L)** Frequency of IFNγ+ cells (out of total cells) broken down by subset between granulomas from D-1MT-treated RMs and control RMs. Subset are ordered as in I. All p-values were calculated with a Wilcoxon Rank Sum test (ns $p > 0.05$, **$p < 0.01$).

Figure S5

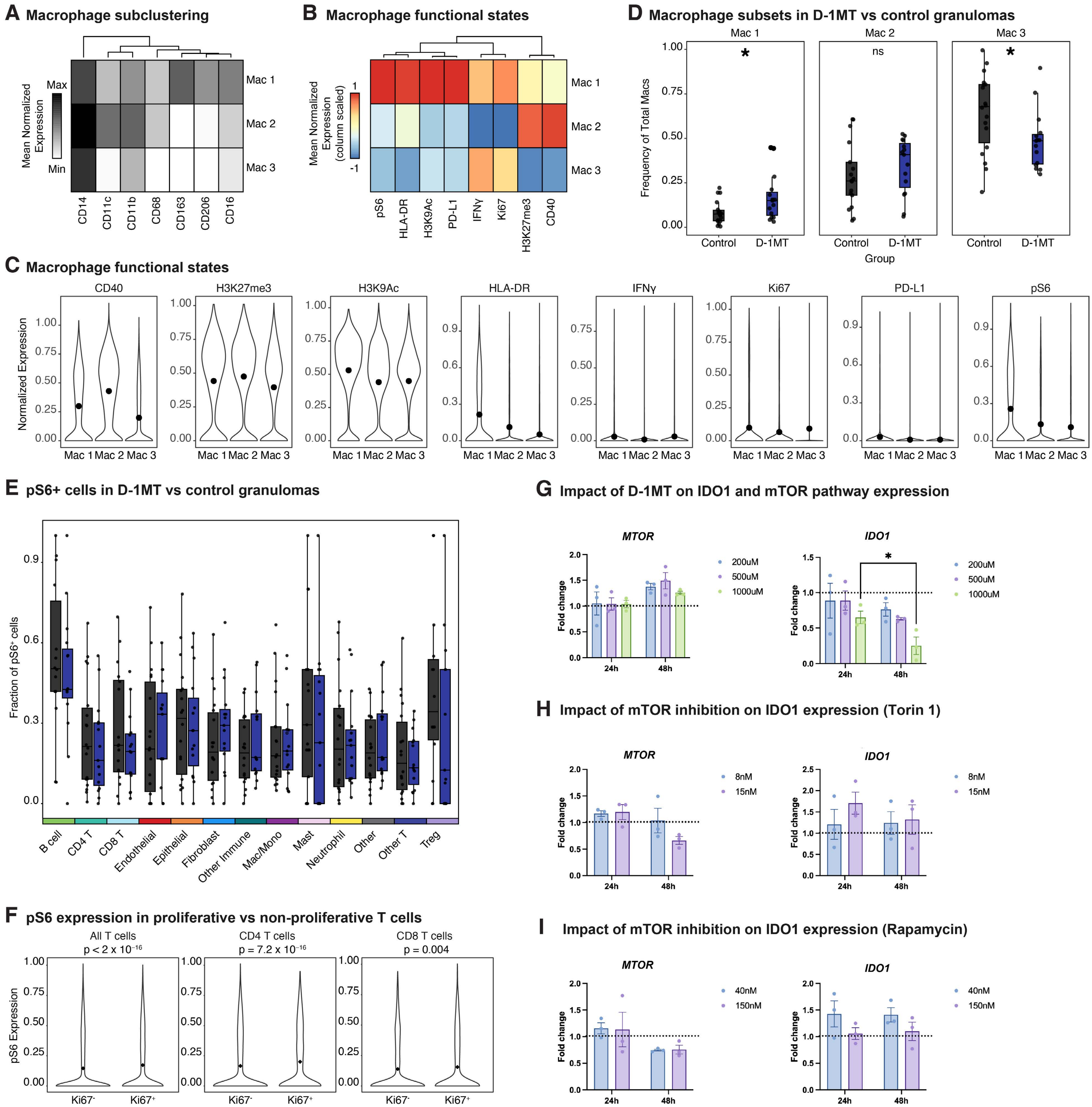

A Macrophage subclustering
Mean Normalized Expression
Max
Min
Mac 1
Mac 2
Mac 3
CD14
CD11c
CD11b
CD68
CD163
CD206
CD16
B Macrophage functional states
Mean Normalized Expression (column scaled)
1
-1
pS6
HLA-DR
H3K9Ac
PD-L1
IFNγ
Ki67
H3K27me3
CD40
D Macrophage subsets in D-1MT vs control granulomas
Frequency of Total Macs
Control
D-1MT
Group
ns
*
C Macrophage functional states
Normalized Expression
CD40
H3K27me3
H3K9Ac
HLA-DR
IFNγ
Ki67
PD-L1
pS6
E pS6+ cells in D-1MT vs control granulomas
Fraction of pS6+ cells
B cell
CD4 T
CD8 T
Endothelial
Epithelial
Fibroblast
Other Immune
Mac/Mono
Mast
Neutrophil
Other
Other T
Treg
F pS6 expression in proliferative vs non-proliferative T cells
All T cells
p < 2 x 10^-16
CD4 T cells
p = 7.2 x 10^-16
CD8 T cells
p = 0.004
pS6 Expression
Ki67-
Ki67+
G Impact of D-1MT on IDO1 and mTOR pathway expression
MTOR
IDO1
Fold change
200uM
500uM
1000uM
24h
48h
H Impact of mTOR inhibition on IDO1 expression (Torin 1)
8nM
15nM
I Impact of mTOR inhibition on IDO1 expression (Rapamycin)
40nM
150nM

Figure S5: Evaluation of macrophage sub-clustering and the mTOR pathway. (A) Heatmap representation of lineage marker expression (columns) across macrophage subclusters (rows) showing the mean normalized expression per marker. **(B)** Expression of functional markers (columns) across macrophage subclusters (rows, ordered as in A) showing the mean normalized expression of each marker scaled by column. **(C)** Violin plot representation of the data shown in B. Black dots represent the mean. **(D)** Frequency of each macrophage subcluster (of total macrophages) in granulomas from control versus D-1MT-treated RMs. **(E)** Frequency of pS6+ cells (out of total cells) broken down by subset between granulomas from D-1MT-treated RMs and control RMs. **(F)** Expression of pS6 in Ki67- versus Ki67+ total T cells (left), CD4+ T cells (middle), and CD8 T cells (right). Black diamond represents the mean expression. All p-values were calculated with a Wilcoxon Rank Sum test (ns $p > 0.05$, *$p < 0.05$). **(G-I)** Expression of *MTOR* (left) and *IDO1* (right) D-1MT **(G)**, Torin 1 **(H)**, or rapamycin **(I)** treated BMDMs at 24- or 48-hours following *Mtb* infection. P-values were determined with a two-way ANOVA.

Figure S6

A Cellular infilatration across granuloma region

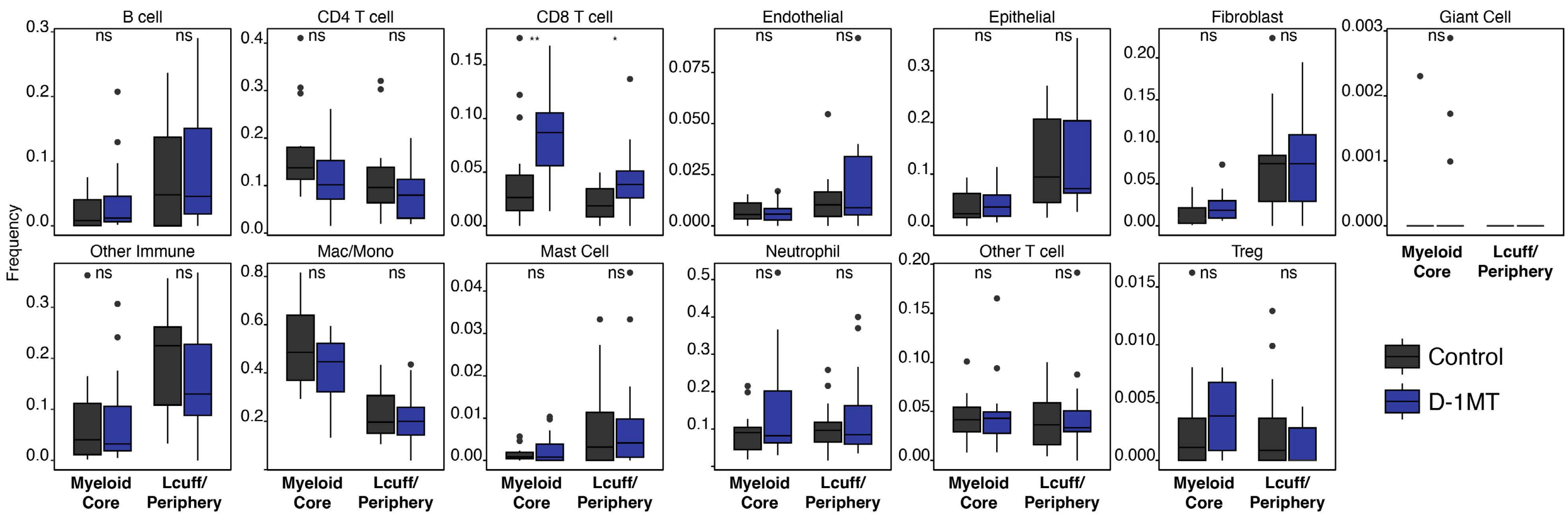

B Pairwise enrichment D-1MT vs Control

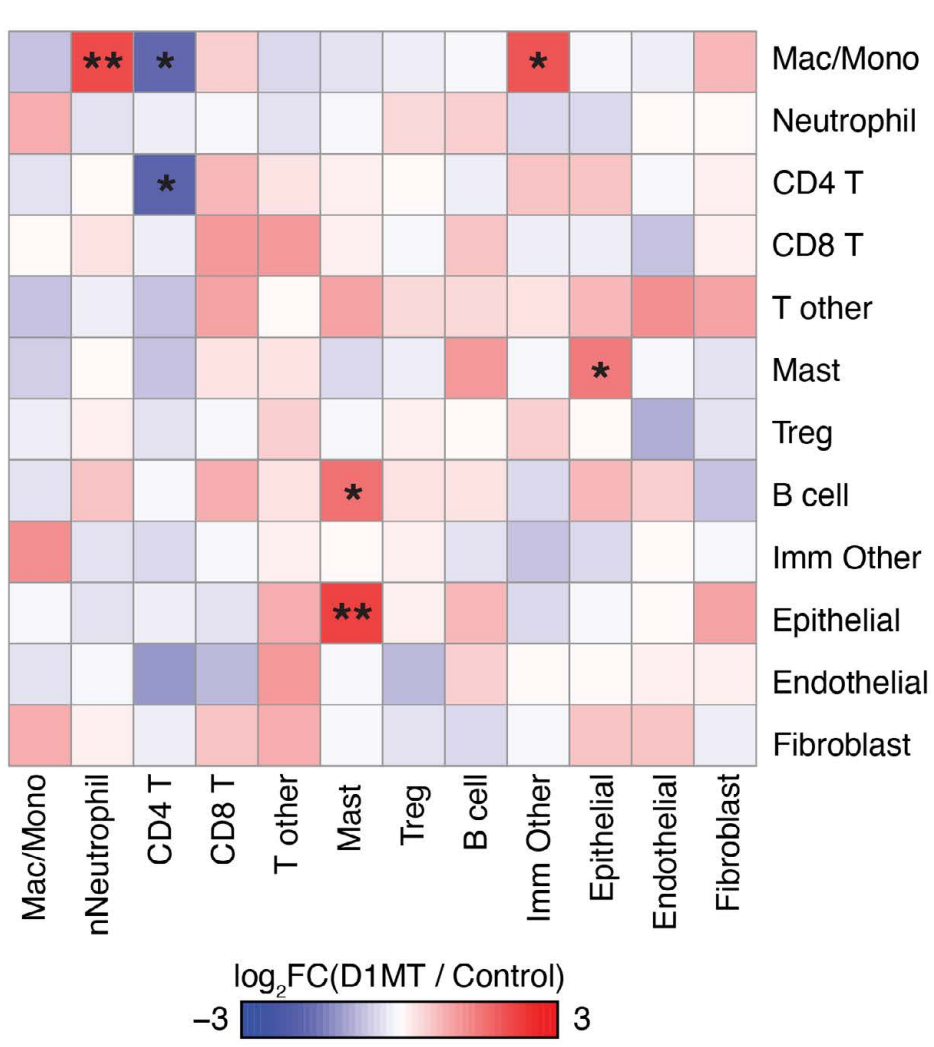

Figure S6: Spatial enrichment of immune cell subsets within RM granulomas. (A) Quantification of the local frequency of each immune cell subset (of total cells) in the myeloid core or lymphocytic cuff (Lcuff)/periphery, also stratified by treatment group. P-values were calculated with a Wilcoxon Rank Sum test (ns $p > 0.05$, **$p < 0.01$). **(B)** Pairwise cell subtype enrichment (or depletion), shown as a heatmap, considering the effect of D-1MT treatment by taking the log2 fold change of D-1MT and control FOVs. P-values were calculated with a Wilcoxon Rank Sum test (*$p < 0.05$, **$p < 0.01$).

Table S1

Day 1 (overnight stain)								Parameters for Analysis	
Antibody Target	Biological Relevance	Provider	Catalog Number	Lot	Clone	Mass Channel	Titer (µg/mL)	Start	Stop
Chymase	Mast cells	Abcam	ab233729	GR32553931	EPR13136	71Ga	0.25	70.7	71
Tryptase	Mast cells	Abcam	ab181724	GR32471283	EPR9522	71Ga	0.25	70.7	71
CD40	Costimulation	Abcam	ab13545	GR322200511	Polyclonal	142Nd	1.00	141.7	142
CD4	T cells	IonPath	714301-100	19147-03	EPR6855	143Nd	1.00	142.7	143
CD14	Monocytes/Macrophages	Cell Signaling Technology	56082BF	2	D7A2T	144Nd	0.50	143.7	144
Foxp3	Regulatory T cells (Tregs)	BD Biosciences	624084	8353822	236A/E7	146Nd	1.00	145.7	146
CD31	Endothelium	Abcam	ab216459	GR32965961	JC/70A	148Nd	0.50	147.7	148
GrzB	Cytotoxic cells	IonPath	715002-100	19235-13	D6E9W	150Nd	1.00	149.7	150
CD209	Myeloid-lineage cells	BD Biosciences	624084	9178683	DCN46	152Sm	0.13	151.7	152
CD206	Anti-inflammatory (M2-like) Macrophages	R&D Systems	MAB25341	CEZR0219091	685645	153Eu	0.50	152.7	153
pS6	Activation	Cell Signaling Technology	4858BF	20	D57.2.2E	154Sm	0.25	153.7	154
CD68	Macrophages	Cell Signaling Technology	76437BF	2	D4B9C	156Gd	0.13	155.7	156
CD11c	Myeloid-lineage cells	Abcam	ab212508	GR32258196	ITGAX/1242	157Gd	0.25	156.7	157
CD8	T cells	Cell Signaling Technology	85336BF	4	D8A8Y	158Gd	0.25	157.7	158
CD3ε	T cells	Cell Signaling Technology	8506BF	4	D7A6E	159Tb	0.50	158.7	159
IDO1	Immunoregulation	Abcam	ab245737	GR32593452	SP260	160Gd	0.50	159.7	160
CD163	Anti-inflammatory (M2-like) Macrophages	Cell Signaling Technology	93498BF	2	D6U1J	163Dy	2.00	162.7	163
CD20	B cells	Cell Marque	120M-8-OEm	1908402	L26	164Dy	0.50	163.7	164
CD16	FcγRIII, Myeloid cells and Natural Killer cells	Cell Signaling Technology	24326BF	2	D1N9L	165Ho	1.00	164.7	165
IFNγ	Inflammatory cytokine	Abcam	ab218890	GR32748436	IFNG/466	166Er	1.00	165.7	166
HLA-DR	MHC class II	Abcam	ab7856	GR32955292	CDR/43	167Er	0.50	166.7	167
CD11b	Myeloid-lineage cells	Abcam	ab187537	GR3245661-3	EP1345Y	168Er	0.25	167.7	168
CD45	Immune cells	Cell Signaling Technology	13917BF	7	D9M8I	169Tm	0.50	168.7	169
H3K9Ac	Chromatin accessibility	Cell Signaling Technology	9649BF	12	C5B11	170Yb	0.50	169.7	170
PanCK	Epithelium	ThermoFisher	MS-343-PABX	19.2FZ343X1810A	AE1/AE3	171Yb	1.00	170.7	171
H3K27me3	Chromatin accessibility	Cell Signaling Technology	9733BF	11	C36B11	172Yb	0.25	171.7	172
Calprotectin	Neutrophils	ThermoFisher	MA1-81381	UE2778733	MAC387	174Yb	0.75	173.7	174
Ki67	Proliferation	Cell Signaling Technology	9449BF	11	8D5	175Lu	0.25	174.7	175

Day 2 (1 hour)								Parameters for Analysis	
Antibody Target		Provider	Catalog Number	Lot	Clone	Mass Channel	Titer (µg/mL)	Start	Stop
HH3	Nuclei	Cell Signaling Technology	4499BF	15	D1H2	89Y	2.00	88.7	89
Vimentin	Cytoplasm	Cell Signaling Technology	5741BF	7	D21H3	113In	2.00	112.7	113
SMA	Myofibroblasts	Cell Signaling Technology	19245BF	4	D4K9N	115In	2.00	114.7	115

Table S1: Details of the antibodies used for the various experiments included in this work for MIBI-TOF.
